# Macrophages co-loaded with drug-associated and superparamagnetic nanoparticles for triggered drug release by alternating magnetic fields

**DOI:** 10.1007/s13346-024-01774-9

**Published:** 2025-01-13

**Authors:** Omkar Desai, Sandhya Kumar, Mario Köster, Sami Ullah, Sushobhan Sarker, Valentin Hagemann, Mosaieb Habib, Nicole Klaassen, Silke Notter, Claus Feldmann, Nina Ehlert, Hansjörg Hauser, Dagmar Wirth

**Affiliations:** 1 Model System for Infection and Immunity, Helmholtz Centre for Infection Research, Inhoffenstr. 7, 38124 Braunschweig, Germany; 2 Institute of Inorganic Chemistry, Leibniz University Hannover, Callinstraße 9, 30167 Hannover, Germany; 3 Institute of Inorganic Chemistry, Karlsruhe Institute of Technology (KIT), Engesserstrasse 15, 76131 Karlsruhe, Germany; 4 iBET-Instituto de Biologia Experimental E Tecnológica, Apartado 12, 2781-901 Oeiras, Portugal; 5 Institute of Experimental Hematology, Hannover Medical School, Carl-Neuberg-Str. 1, 30625 Hannover, Germany

**Keywords:** Macrophages, Superparamagnetic iron oxide nanoparticles, Inorganic–organic nanoparticles, Mesoporous silica nanoparticles, Alternating magnetic field, 4-hydroxytamoxifen

## Abstract

Two features of macrophages make them attractive for targeted transport of drugs: they efficiently take up a broad spectrum of nanoparticles (NPs) and, by sensing cytokine gradients, they are attracted to the sites of infection and inflammation. To expand the potential of macrophages as drug carriers, we investigated whether macrophages could be simultaneously coloaded with different types of nanoparticles, thus equipping individual cells with different functionalities. We used superparamagnetic iron oxide NPs (SPIONs), which produce apoptosis-inducing hyperthermia when exposed to an alternating magnetic field (AMF), and co-loaded them on macrophages together with drug-containing NPs (inorganic–organic nanoparticles (IOH-NPs) or mesoporous silica NPs (MSNs)). We show that individual macrophages can take up both SPIONs and drug-loaded NPs efficiently, thereby generating drug-loaded cells susceptible to AMF-induced cell death. Macrophages co-loaded with SPIONs and drug-containing IOH-NPs spontaneously released the drugs at similar rates irrespective of the application of an AMF. Notably, while the spontaneous drug release from macrophages co-loaded with SPIONs and drug-associated MSNs was low, AMF exposure accelerated the drug release. Thus, AMF exposure of SPION/drug-MSN coloaded macrophages provides a simple strategy for trigger-controlled drug release since it does not require any chemical modification of NPs or drugs. Thus, we assume that the coloading of different types of NPs will expand the potential of macrophages for drug delivery.

**Graphical Abstract:**

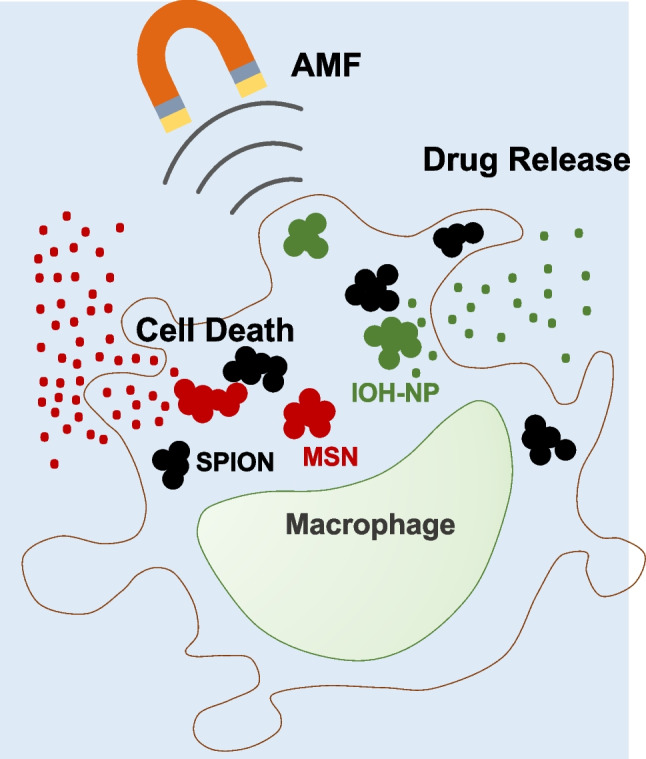

**Supplementary Information:**

The online version contains supplementary material available at 10.1007/s13346-024-01774-9.

## Introduction

In recent decades, nanomaterials with various compositions and shapes have been developed as promising tools for drug delivery in a plethora of medical applications [1]. Depending on the specific design, drugs can be located within cores and coated with shells, or covalently or noncovalently attached to the surface of a scaffold. Furthermore, nanoparticles (NPs) can be functionalized by attaching surface molecules to target specific cell types, facilitating enhanced cellular uptake [1–3] or by integrating magnetic cores that serve as contrast agents for sensitive magnetic resonsance imaging (MRI) applications [4]. However, the combination of various features on individual NPs to create multifunctional NPs is complex and remains a challenge.

The therapeutic application of NPs is also hampered by their limited half-life in circulation, a consequence of their rapid clearance by the host’s reticoendothelial system, which results in a low number of NPs reaching and accumulating at the site of interest [5, 6]. Various strategies have been used to increase NP stability in vivo. An interesting variant that has recently emerged is the coating of NPs with cellular membranes, which not only mimics the surface properties of donor cells, but also increases the circulation lifetime in vivo [7, 8]. This concept was further developed by loading NPs into living cells. Macrophages have been the focus of current research. Based on their ability to take up pathogens and cell debris, they can be efficiently loaded with various types of NPs in vitro [9–11]. Upon transplantation of such loaded macrophages, NPs are protected from immediate clearance and degradation, which improves their stability and prolongs the half-life of their associated drugs in vivo [12, 13]. In addition, since macrophages home across endothelial barriers and accumulate in diseased tissues in response to chemokines/cytokines, they can deliver NPs to tumors or inflamed tissues [9, 14, 15].

Among the various types of NPs considered for therapeutic applications, those used for drug delivery are of particular interest. Mesoporous silica NPs (MSNs) have emerged as promising nanomaterials with high biocompatibility [16]. Owing to their ordered mesopores with large pore volumes (0.6–1 cm^3^/g) and surface areas (700–1000 m^2^/g) [17], these particles can be loaded by diffusion and can hold large quantities of substances. Drug release from MSNs occurs spontaneously, with kinetics depending on the physicochemical properties of the drugs as well as the pore size. Recently, inorganic‒organic NPs (IOH-NPs) became of interest for drug delivery. IOH-NPs are characterized by a saline composition with an inorganic cation and a phosphate-functionalized drug anion. This group of NPs allows an unprecedented drug load of > 70% of the total nanoparticle mass and shows high biocompatibility and biodegradability. This phenomenon has been exemplified by various compounds including 5′-fluoro-2′-deoxyuridine 5′-monophosphate (FdUMP) [18], gemcitabine monophosphate (GMP) [19], betamethasone phosphate (BMP) [20, 21] and clindamycin phosphate (CLP) [22], which are chemotherapeutic, anti-inflammatory, and antibacterial agents.

Superparamagnetic iron oxide NPs (SPIONs) are the focus of clinical applications because of their ability to produce hyperthermia when exposed to an alternating magnetic field (AMF) [23–25]. SPIONs are characterized by superparamagnetic Fe^2+^/Fe^3+^ cores that are stabilized by various biocompatible shells, including silica (SiO_2_), dextran, and polyethylene glycol (PEG), making them resistant to biodegradation and polymerization [26]. SPIONs have attracted interest for multimodal tumor therapy [27]. SPIONs generate mechanical forces when exposed to AMF at a low frequency, thereby inducing destructive effects on tumor tissues [28]. However, after injection into the tumor tissue and high-frequency AMF application, hyperthermia occurs (at 43–46 °C), which is sufficient to induce apoptosis or necrosis of neighboring (cancer) cells without harmful effects at greater distances [29, 30]. This method has recently entered clinical trials for the killing of tumor cells for anticancer treatment, either alone or in combination with conventional chemotherapy or radiotherapy [31]. Recently, we demonstrated that SPIONs are efficiently taken up by macrophages [32]. In 3D cocultures of SPION-loaded macrophages and tumor cells (spheroids), we observed that exposure to AMF induced specific killing of the loaded macrophages, which was associated with membrane disruption and release of SPIONs, while non-loaded cells remained intact.

In this study, we evaluated whether the co-loading of different types of NPs on macrophages is possible without compromising their particular features. We demonstrate efficient co-loading of drugs containing NPs (clindamycin phosphate (CLP)-loaded [ZrO]^2+^[CLP]^2–^ IOH-NPs (ZrO-CLP-NPs) [22] or 4-hydroxytamoxifen (4-OHT)-loaded MSNs) with SPIONs. AMF exposure of co-loaded macrophages resulted in efficient cell death of carrier cells, whereas neighboring cells were not affected. The drug release kinetics of the ZrO-CLP-NPs was not affected by the induction of hyperthermia in the co-loaded macrophages. AMF exposure upon 4-OHT-MSN/SPION co-loading resulted in increased release of 4-hydroxytamoxifen, Endoxifen, and Raloxifene. In summary, co-loading of macrophages with NPs of different natures is an efficient method for the simultaneous delivery of various therapeutic agents to diseased tissues.

## Results

### The properties of IOH-NPs and SPIONs are maintained within co-loaded macrophages

To investigate if different NPs can be co-loaded onto macrophages without compromising their specific features we selected AMF-responsive SPIONs and drug-containing NPs. For the latter, we focussed on MSNs and ZrO-based IOH-NPs, since they can incorporate drugs of various natures without the need for additional chemical modifications. The features of the different NPs are summerized in the Supporting Information A and B.

**Fig. 1:**
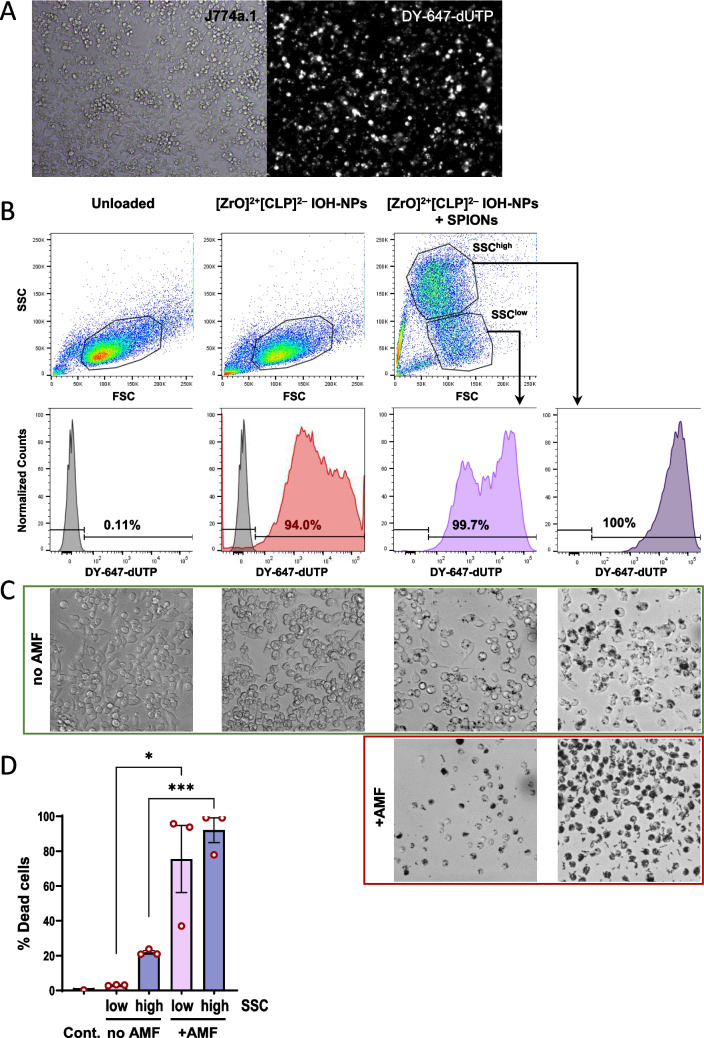

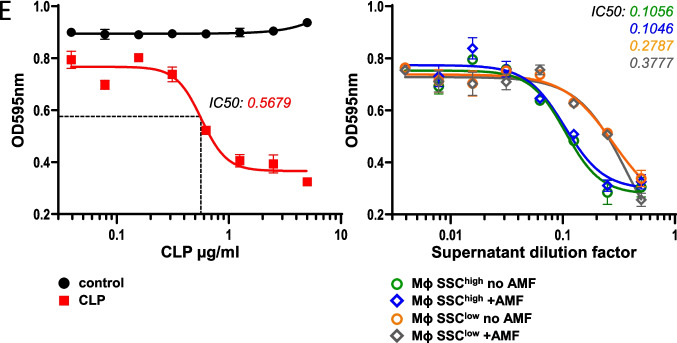
Coloading of SPIONs and cargo NPs. **A** Representative fluorescence microscopy pictures of J774a.1 macrophages loaded with ZrO-CLP-NPs labelled with the fluorescent dye DY-647-dUTP (10 × objective, Zeiss filter set Rhod Ex 545/25, Em 606/70). **B** Forward (FSC) and side (SSC) scatter properties of J774a.1 macrophages, either non-loaded, loaded with ZrO-CLP-NPs or co-loaded with ZrO-CLP-NPs and SPIONs (upper panel). Number and fluorescence intensity (APC channel, excitation with 640 nm, emission collected with bandpass filter 670/30 nm) of differentially loaded cells, indicating uptake of DY-647-dUTP-labled ZrO-CLP-NPs in single and co-loaded cells as well as in sorted SSC^high^ and SSC^low^ populations (lower panel). **C** Representative brightfield microscopic images of sorted SSC^high^ and SSC^low^ cell populations indicated in (**B**) 24 h after exposure to AMF (+ AMF, highlighted by green frame) or mock treated (no AMF, red frame) (image acquisition with 20 × objective). **D** Macrophages co-loaded with ZrO-CLP-NPs and SPIONs were subjected to AMF exposure or mock treatment. Cells were re-seeded, cultivated for 24 h and stained with the cell death marker Nuclear Green DCS1. Flow cytometry was used to gate into SSC^low^ and SSC^high^ populations and the frequency of Nuclear Green DCS1-labeled death cells was determined for the individual populations (bl525 channel, excitation with 488 nm, emission collected with bandpass filter 525/50 nm). Non-loaded cells stained with Nuclear Green DCS1 was used as control. Values represent means ± SEM for percent of Nuclear Green DCS1-positive cells (*n* = 3). * = *P* < 0.05; *** = *P* < 0.001. **E** Light scatter absorbance at 595 nm of bacterial cultures (indicating bacterial density) after exposure to serial dilutions of free CLP (5 µg/ml to 0.039 µg/ml) (left graph) or culture supernatants from sorted SSC^high^ and SSC^low^ populations harvested 24 h after AMF exposure or mock treatment (right graph). IC50 value of CLP activity was calculated from a nonlinear regression with four parameters (GraphPad Prism). IC50 values for the macrophage supernatants (depicted in the right graph) represent dilution factor to obtain half-maximal inhibition of bacterial growth

We examined the ability of macrophages to take up both ZrO-CLP-NPs and SPIONs. To this end, we employed a sequential loading protocol. J774a.1 macrophages were first incubated with DY-647-labelled ZrO-CLP-NPs containing the antibiotic CLP [22, 33]. After excessive washing to remove extracellular particles, microscopic examination demonstrated efficient uptake of fluorescently labled NPs at the cell population level (Fig. 1A). Flow cytometry analysis of these cells indicated strongly increased fluorescence intensity in the APC (DY-647) channel, demonstrating high and homogenous uptake efficiencies for ZrO-CLP-NPs in the entire cell population (Fig. 1B), without changes in cell morphology (Fig. 1C). Subsequently, the cells were exposed to SPIONs. SPION loading altered the internal complexity of the cells. This could be visualized by flow cytometry as a consequence of altered laser light reflection or refraction at the intracellular SPIONs, resulting in an overall increase in the side scatter (SSC) signal (Fig. 1B). The SSC properties of the cells were used to sort the two populations. Both the SSC^low^ and SSC^high^ populations showed high DY-647 fluorescence intensities, demonstrating efficient co-loading with the ZrO-CLP-NPs and SPIONs. Interestingly, the SSC^high^ population with maximal SPION content also demonstrated a higher DY-647 fluorescence, suggesting that a proportion of cells had higher uptake efficiencies for both types of NPs. We confirmed that the sorted cell populations were co-loaded with ZrO-CLP-NPs/SPIONs by microscopy image analysis. SPION loading was accompanied by an increase in cell refractions in the brightfield images and was more homogenous in the SSC^high^ cell population (Fig. 1C). Nevertheless, both the SSC^low^ and SSC^high^ populations showed intracellular accumulation of SPIONs, whereas the SSC^high^-gated cells contained a large proportion of cells with greater uptake efficiency for both types of NPs (Fig. 1B, C).

Previously, we demonstrated that hyperthermia induced upon AMF exposure of SPION-loaded J774a.1 cells is sufficient to kill carrier cells [32]. To investigate whether AMF responsiveness is maintained after macrophage co-loading, we investigated whether AMF-exposed SSC^low^ and SSC^high^ populations undergo cell death. Co-loaded cells were subjected to AMF for 20 min and re-seeded in standard cell culture medium. Microscopic inspection of both sorted populations 24 h after AMF exposure indicated a change in cell morphology and SPION localization compared to control cells. While in mock-treated cells SPION localization was characterized by small punctate spots as well as some larger aggregates, AMF exposed cells displayed mainly aggregates of intracellular SPIONs, in addition to the emergence of extracellular fiber-like SPION structures (Fig. 1C). These alterations were much more pronounced for the SSC^high^-gated population. Notably, we also observed cell death in the SSC^low^-gated population, characterized by rounded, opaque cells, indicating that a low SPION load was sufficient to induce hyperthermia (Fig. 1C). To quantify AMF-induced cell death, we made use of the cell death indicator Nuclear Green DCS1. This membrane-impermeable DNA-binding dye cannot enter living cells and thus discriminates between living and dead cells. Thus, 24 h after AMF or mock treatment, we quantified the labelled dead cells by flow cytometry. Figure 1D indicates that the SSC^high^ population comprised a small proportion of dead cells during mock cultivation. However, the frequency of dead cells in SSC^low^ and SSC^high^ populations strongly increased from 3.1% to 75.5% and from 21.9% to 92.1% upon AMF exposure, respectively. Thus, AMF exposure efficiently kills double loaded cells.

Next, we investigated whether the induction of hyperthermia modulates the release kinetics of ZrO-CLP-NP-loaded cells. To this end, we sequentially co-loaded J774a.1 cells with both ZrO-CLP-NP and SPIONs according to the protocol described above. We isolated SSC^low^ and SSC^high^ populations by cell sorting, subjected both cell populations to AMF or mock treatment and subsequently cultured all groups in standard conditions. After 24 h, we collected the supernatants of the SSC^low^ and SSC^high^-sorted cell populations. The antimicrobial effect of the supernatants was measured on exponentially growing *S. aureus*. The results showed efficient growth inhibition of *S. aureus* in supernatants from both AMF-exposed and non-exposed cells (Fig. 1E). Serial dilutions of the supernatant revealed a half-maximal inhibition of *S. aureus* growth at dilution factors of 0.27 and 0.1, corresponding to a concentration of 2.1 and 5.7 µg/ml CLP, for SSC^low^ and SSC^high^ cells, respectively. This reflects the different NP loading efficiencies of the two populations. Importantly, the antimicrobial activity of the supernatants did not change upon AMF exposure of cells. This indicates that hyperthermia did not affect the release of clindamycin, suggesting that the dissolution of ZrO-CLP-NPs occurred with comparable efficiency in the absence and presence of hyperthermia. Overall, we showed that J774a.1 macrophages can be efficiently loaded with ZrO-CLP-NPs and SPIONs without compromising hyperthermia induction or drug release.

### Phenotype of MSN/SPION co-loaded macrophages and responsiveness to AMF

**Fig. 2:**
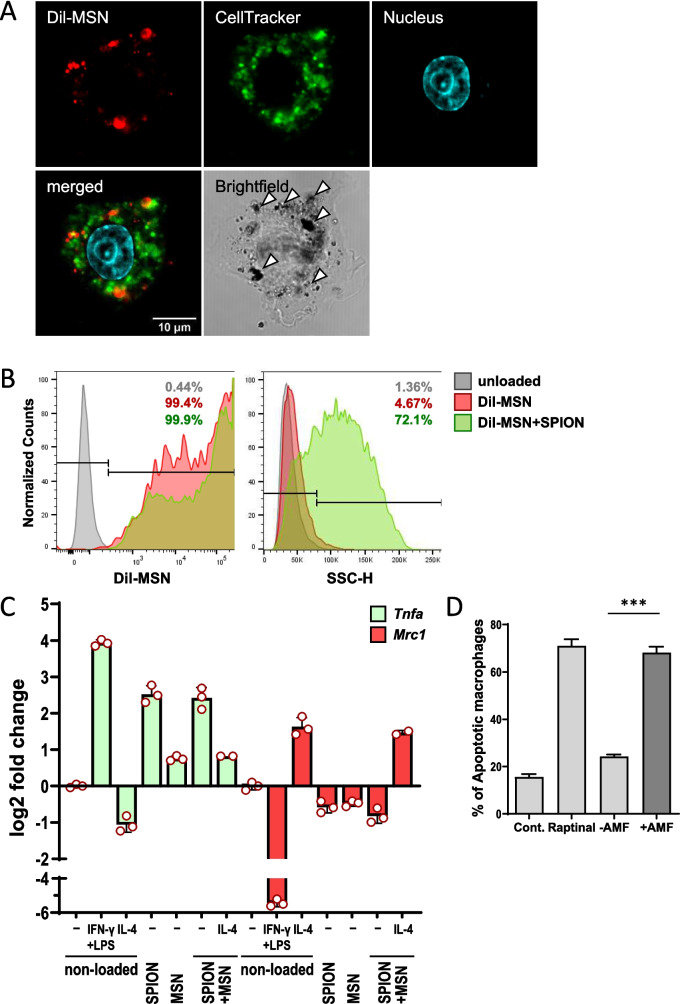
Intracellular uptake of the SPIONs and cargo-loaded MSNs. **A** Representative confocal fluorescence microscopy pictures of J774a.1 cells co-loaded with fluorescently labelled Dil-MSNs and SPIONs. For visualization, the cells were costained with Cell Tracker Green Stain (Ex 488 nm) and Hoechst 33,258 (Ex. 405 nm). Brightfield image visualizes uptake of SPIONs (black clusters indicated by white triangles). **B** Fluorescence intensities (PE channel, Ex 561 nm, Em BF 582/15 nm) and side scatter properties of Dil-MSN/SPION-coloaded J774a.1 cells as obtained by flow cytometry. Representative histograms indicate loading efficiencies of 99% for Dil-MSNs and 72% for co-loading with SPIONs. Unloaded cells or J774a.1 cells incubated with only Dil-MSNs are depicted as controls. **C** BMDMs were either loaded with SPIONs or MSNs or co-loaded and cultivated for 24 h in standard culture conditions. Non-loaded BMDMs were incubated for 24 h either with control medium, with IFN-γ (50 ng/ml) and LPS (100 ng/ml) or with IL-4 (20 ng/ml). Besides, MSN/SPION-co-loaded macrophages were treated with IL-4 (20 ng/ml) for 24 h. mRNA expression of M1 (*Tnfa*) and M2 (*Mrc1*) markers was determined by RT-qPCR and normalized to beta-actin expression. Results are expressed as means S.D. (*n* = 3). **D** The fraction of apoptotic cells was quantified 24 h after AMF- or mock-exposure of MSN/SPION-coloaded BMDMs by Annexin V-staining and flow cytometry analysis (FITC channel; excitation with 488 nm, emission collected with bandpass filter 525/50 nm). Unloaded cells or cells treated with Raptinal were used as controls. Values represent means ± SEM for percent of Annexin V-positive cells (*n*= 3). *** = *P* < 0.001

We evaluated whether MSNs could be used as drug carriers and co-loaded with SPIONs. To track the intracellular localization of the co-loaded MSNs, we labelled MSNs with the lipophilic carbocyanine (red) fluorescent dye Dil (Dil-MSNs) and sequentially loaded these particles and SPIONs onto J774a.1 cells as described above. The uptake of the Dil-containing MSNs was verified using confocal microscopy. Red fluorescent spots and black structures within the cells in the bright-field image demonstrate the incorporation of MSNs and SPIONs, respectively. Interestingly, MSNs and SPIONs were found at distinct intracellular sites in the cells, indicating that they were localized to different compartments (see Fig. 2A for a representative image). We quantified the percentage of cells after loading with the Dil-labelled MSNs or after sequential loading of the Dil-MSNs and SPIONs using flow cytometry (Fig. 2B). Notably, macrophages showed a high loading efficiency for MSNs (99% of the exposed cells), as indicated by the single loading of DiI-labelled particles. Quantification of fluorescence intensity revealed the uptake of about 61 pg MSNs per cell (Supporting Information C1). We assessed the co-loading efficiency by flow cytometry, using the SSC properties of the cells as a measure of SPION uptake and the fluorescence of cells as a measure of Dil-MSN uptake. In contrast to the single MSN-loaded population, an increased side-scatter signal was observed for the population loaded with both types of NPs, indicating a co-loading efficiency of at least 70% (Fig. 2B).

A hallmark of cells of the monocyte-macrophage lineage is the ability to differentially respond to environmental cues by adopting distinct functional phenotypes, so-called classical (M1) or alternative (M2) polarized states. We asked if loading of the various NPs alone or in combination would induce macrophage polarization and quantified the mRNA levels of the M1 marker gene *Tnfa* and the M2 marker gene *Mrc1* in differently loaded macrophages. As control, we stimulated non-loaded macrophages with LPS/IFN-γ or IL-4 to trigger an inflammatory M1-like phenotype or an anti-inflammatory M2-like phenotype, respectively. While polarization of non-loaded macrophages to M1 and M2 resulted in specific upregulation of *Tnfa* and *Mrc1*, respectively, loading with MSNs or ZrO-CLP-NPs alone did not change expression of these marker genes (Fig. 2C; Supporting Information C2).

In contrast, loading with SPIONs as well as SPIONs plus MSNs induced expression of the M1 marker *Tnfa* but not the M2 marker *Mrc1*, indicating that SPION loading shifts the cells to an inflammatory-like phenotype. We asked if loaded cells with an M1 phenotype are still capable to respond to environmental cues. Thus, we challenged SPION/MSN co-loaded cells with IL-4 and analyzed for *Tnfa* and *Mrc1* gene expression. We observed that IL-4 stimulation significantly reduced *Tnfa* expression in these cells, while it increased *Mrc1* expression (Fig. 2C). Futher, we observed only marginal production of type I interferons (IFNs) upon loading macrophages with MSNs or ZrO-CLP-NPs alone (Supporting Information C3), suggesting that uptake of these NPs led to minimal stimulation of the innate immune pathway. This demonstrates that the double-loaded cells are capable to activate the specific intracellular signaling pathways and can be reprogrammed, similar to non-loaded cells.

To determine the efficiency of AMF responsiveness, we measured the cell death of AMF-exposed macrophages sequentially loaded with MSNs and SPIONs using an annexin V-based apoptosis assay. AMF exposure of MSN/SPION co-loaded cells showed efficient cell death, while the viability of the non-exposed loaded cell population was similar to that of the non-loaded macrophages (Fig. 2D). In conclusion, we demonstrated efficient co-loading of macrophages with MSNs and SPIONs without compromising the cellular plasticity and confirmed the response of SPION-loaded cells to AMF.

### Accelerated drug release upon AMF exposure of 4-OHT-MSN/SPION co-loaded macrophages

**Fig. 3:**
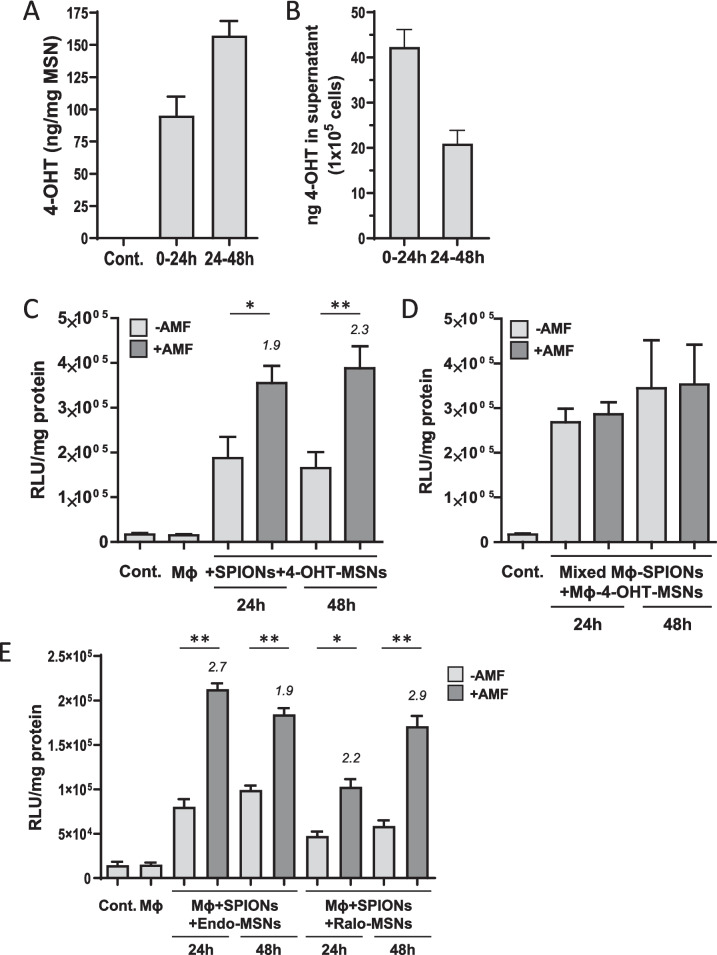
AMF exposure increases cargo release from MSN/SPION co-loaded macrophages. **A** Kinetics of 4-OHT release from cell-free loaded MSNs. 4-OHT activity in the supernatant of 80 µg/ml loaded MSNs was determined after 24 h and after the following 24 h of incubation at 37 °C. Values were normalized to 1 mg MSNs and represent means ± SEM (*n* = 9). **B** Quantification of spontaneously released 4-OHT during cultivation of MSN-loaded BMDMs. After loading with 4-OHT-MSNs, BMDMs were washed three times. Supernatants were collected after 24 h (0–24 h) and after the following 24 h cultivation period (24–48 h). Supernatants were diluted and transferred to Cre-luc reporter cells. Luciferase activity was normalized to ng 4-OHT release into total supernatant and 1 × 10^5^ cells. Values indicate mean ± SEM (*n* = 3). **C** 4-OHT release from BMDMs co-loaded with 4-OHT-MSNs and SPIONs 24 h and 48 h after AMF exposure or mock treatment. Macrophages were co-cultured with Cre-luc reporter cells using transwell inserts. The control (Cont.) indicates background of the assay and Mϕ indicates luciferase activity of reporter cells in presence of unloaded BMDMs. Values represent means ± SEM of relative light units (RLU) normalized to the protein content of reporter cell lysate (*n* = 6). Numbers above bars indicate fold change compared to corresponding mock-treated conditions. * = *P* < 0.05, ** = *P* < 0.01. **D** AMF-induced 4-OHT release from 1:1 mixtures of BMDMs independently loaded with either 4-OHT-MSNs or with SPIONs. Macrophages were co-cultured with Cre-Luc reporter cells using transwell inserts. The control (Cont.) indicates the background of assay. Values represent means ± SEM of relative light units (RLU) normalized to the protein content of reporter cell lysate (*n* = 3). **E** Kinetics of drug release from BMDMs sequentially co-loaded with Endoxifen-MSNs (Endo) or Raloxifene-MSNs (Ralo) and SPIONs after AMF exposure or mock treatment as described in (**C**). Values represent means ± SEM of relative light units (RLU) normalized to the protein content of reporter cell lysate (*n* = 3). Numbers above bars indicate fold change compared to corresponding mock-treated conditions. * = *P* < 0.05, ** = *P* < 0.01

To determine cargo release from MSN/SPION co-loaded macrophages, we used 4-OHT as a model drug. 4-OHT is an antiestrogen binding to the estrogen receptor α and is frequently used in breast cancer therapy. 4-OHT-loaded MSNs were generated by incubating MSNs in H_2_O supplemented with 4-OHT (see Materials and methods for details). First, we determined the spontaneous release of the cargo from the 4-OHT-MSNs in a cell-free environment. 4-OHT-MSNs were incubated in the cell culture medium at 37 °C. After 24 h, the samples were centrifuged and the supernatant was collected. The particles were resuspended in fresh culture media and incubated for another 24 h. The amount of 4-OHT in the supernatants of MSNs was determined using a functional reporter cell assay which provides a sensitivity of 2 ng/ml (Figure S15). In a cell-free environment, we detected a continuous release of 4-OHT from 4-OHT-MSNs over time, resulting in 95 ng and 157 ng 4-OHT/mg MSN after incubation from 0–24 h and 24-48 h, respectively (Fig. 3A). Next, we measured the spontaneous release of 4-OHT from 4-OHT-MSN-loaded macrophages into the cell supernatant over a period of 48 h. 1 × 10^5^ BMDMs released 42.3 ng and 21 ng 4-OHT at 24 h and 48 h, respectively, indicating the spontaneous release of 4-OHT from MSNs and diffusion out of the cells (Fig. 3B).

Finally, we determined whether AMF-induced hyperthermia could modulate the kinetics of 4-OHT release. BMDMs were subjected to sequential co-loading with 4-OHT-MSNs and SPIONs. The co-loaded cells were then exposed to AMF for 20 min. To monitor the release of biologically active 4-OHT over time and to mimic a therapeutic setting, we used transwell co-cultures of co-loaded macrophages and 4-OHT responding reporter cells (Fig. 3C). Mock-treated cells were used as controls to detect spontaneous release of 4-OHT. While macrophages were seeded into transwell insert, Cre-luc reporter cells were co-cultured at the bottom of the cell culture plate and luciferase activity was determined 24 h and 48 h after co-culture. The co-culture of loaded, mock-treated BMDMs with reporter cells increased the expression of luciferase up to 11-fold, indicating spontaneous release of biological active 4-OHT out of the cells. Notably, AMF exposure of co-loaded macrophages increased 4-OHT release by 1.9-fold and 2.3-fold at 24 and 48 h, respectively, compared to mock-treated cells (Fig. 3C). Since without AMF exposure, the 4-OHT activities in the supernatant were comparable at 24 h and 48 h, we conclude that only a portion of 4-OHT was released during cultivation of intact macrophages, while the rest was retained within the cells. However, AMF exposure boosted the release of 4-OHT at both time points.

To confirm whether co-loading with both types of NPs is necessary to achieve an enhanced release of 4-OHT upon AMF exposure, we loaded the macrophages separately with SPIONs or 4-OHT-MSNs according to the protocol specified above. Then, differentially loaded cells were mixed and exposed to AMF. Quantification of the 4-OHT activity in the supernatants showed comparable levels for AMF-treated and mock-treated mixed cells, both at 24 h and at 48 h (Fig. 3D). This demonstrates that AMF-induced hyperthermia acts locally within the SPION carrier cells, but has no effect on 4-OHT-MSN-loaded neighboring cells.

Further, we investigated whether loading MSNs with other inducers of Cre-ERT2 activity recapitulated the release of 4-OHT from MSNs taken up by macrophages. Thus, we loaded MSNs with Endoxifen or Raloxifene, two 4-OHT analogs that bind to estrogen receptor alpha (ERα) at nanomolar concentrations [34]. Notably, the release properties of MSNs loaded with Raloxifene under cell free conditions were consistent with those of 4-OHT, although slightly lower biological activity was observed (data not shown). Additionally, spontaneous and hyperthermia-induced release followed similar kinetics, as indicated by the 1.9- to 2.9-fold increase in luciferase expression compared with that induced by mock-treated cells (Fig. 3E).

Together, these results demonstrate that the co-loading of drug-containing MSNs and SPIONs on macrophages triggers enhanced drug release upon AMF exposure.

## Discussion

The inherent properties of macrophages make them attractive tools for drug delivery, particularly for local diseases, such as cancer or infections [9, 35]. Macrophages, endowed with the natural ability to engulf foreign entities, serve as ideal carriers for drug-loaded NPs, thereby preventing their immediate clearance from the circulation. Moreover, the ability of macrophages to sense pathological cues and migrate selectively towards gradients of cytokines and chemokines provides the opportunity to target NPs to sites of infection or inflammation [36, 37].

In this study, we investigated whether macrophages can be equipped with novel functions upon co-loading with different types of NPs. We demonstrated that macrophages can be efficiently co-loaded with AMF-responsive SPIONs as well as with drug-containing NPs, such as IOH-NPs or MSNs, thereby transferring the properties of both types of NPs to a single macrophage. In our study, the release of drugs from drug-loaded NPs was combined with the AMF-responsiveness of SPIONs to damage and destroy the carrier macrophages.

Notably, responsiveness to AMF was maintained, as reflected by AMF-dependent hyperthermia, which was sufficient to efficiently kill the loaded cells under both co-loading conditions. Additionally, the release of biologically active drugs from the ZrO-CLP-NPs and MSNs did not decrease after co-loading. In contrast, we observed increased drug release when 4-OHT-MSN/SPION-coloaded macrophages were exposed to AMF. We hypothesized that hyperthermia-induced apoptosis, which is accompanied by the disintegration of cellular membranes as well as increased drug mobilization of 4-OHT from 4-OHT-MSNs due to elevated temperatures in the phagolysosomal compartment, may contribute to this effect. Importantly, the effect of hyperthermia was highly localized, as the close proximity of the SPIONs and 4-OHT-MSNs within the same cell was critical for AMF-triggered increases in drug release. We suggest that the co-loading strategy and AMF-induced release could induce drug release at a specific time point, leading to a triggered increase in the amount of loaded drugs rather than a delayed continuous release.

The uptake of MSNs or ZrO-NP did not lead to macrophage polarization, suggesting that it does not have a major impact on the cellular features. In contrast, SPION-loading induced an M1-like phenotype. Importantly, the fate of SPION-loaded cells could be reverted by exposing them cells to IL-4, an inducer of the M2 state. This indicates that even the SPION-loaded cells are not terminally fixed but rather respond to environmental cues. This feature is of particular relevance for in vivo application of cells.

In a previous study, we loaded macrophages with conjugated NPs, in which a toxin was covalently bound to SPIONs via a thermolabile linker. When these cells are exposed to AMF, hyperthermia leads to the apoptosis of macrophages and triggers toxin release from SPION conjugates [32]. While covalent linkage of the toxin impairs spontaneous drug release, the generation of covalently linked drug-SPION conjugates is usually demanding because the synthesis of such conjugates can be complex depending on the chemical nature of the drug. In this study, we achieved AMF-induced drug release by coloading AMF-sensitive SPIONs and drug-MSN complexes in individual macrophages, without the need for additional chemical modification. Therefore, this method is of interest for the triggered release of various drugs. Nevertheless, we anticipate that the applicability depends on the intrinsic properties of the drug and MSNs, such as conformation, adhesion force, and pore size. Here, we demonstrated that not only 4-OHT but also its derivatives, Raloxifene and Endoxifen, fulfil these requirements. For other applications, identification of suitable drug/MSN combinations is required.

IOH-NPs have been shown to be widely applicable for diagnosis and therapy in medicine and molecular biology [38]. Owing to their composition, they can accommodate very high drug loads, making them attractive drug delivery systems. The ZrO-CLP-NPs used in this study were characterized by rapid degradation within the cellular environment due to the presence of ubiquitous classes of intracellular phosphatases that are expected to support the degradation of these particles. We suggest that additional modifications of NP composition could further delay drug release and make these particles suitable for SPION/AMF-triggered drug release. Similarly, an appropriate coating of drug-soaked MSNs with a shell that reduces spontaneous release may further improve hyperthermia-triggered release. In this regard, it would be tempting to take advantage of the specific intracellular conditions, such as the low pH that prevails in the lysosomal compartments of carrier macrophages, but also other physiological features that change upon time.

Patient-specific macrophages can be purified from blood and can also be generated upon differentiation of induced pluripotent stem cells in large numbers [39, 40]. This paves the way for therapeutic applications. So far, macrophage-based clinical trials focuss on therapy of cancer and cardiomyopathies [41], while the use of macrophages for drug delivery is a more recent field of research [9, 13, 15, 42]. From a theoretical point of view, the exploitation of phagocytic cells for the delivery of drugs would provide a number of advantages: by avoiding systemic drug delivery and by site-specific drug release side effects arising from deleterious adverse reactions in off-target tissues can be expected to be overcome or at least reduced. Moreover, within the macrophages, drug-associated NPs are exposed to a controlled intracellular environment, largely shielded from physiological changes in tissue or body fluids and protected from degradation and clearance. Finally, the targeted delivery allows to substantially decreases the amount of drugs applied. On the other hand, macrophage-based drug delivery would need to comply with safety, logistics and regulatory requirements. Thus, macrophage-based delivery routes are foreseen for hard-to treat diseases, which e.g. require the application of drugs with high systemic toxicity.

## Conclusions

Coloading with different types of NPs is efficient and can be used to combine different NP properties in single macrophages, thereby extending the potential of these cells as drug carriers. The combination of SPIONs and drug-loaded MSNs in single macrophages enables the controlled release of drugs upon exposure to AMF, and paves the way for the triggered release of drugs upon macrophage-mediated transport to inflamed sites. Future developments are expected to provide alternative NP combinations for macrophage functionalization, opening further perspectives in the emerging field of macrophage-based therapies.

## Materials and methods

### Synthesis of DY-647-labelled ZrO-CLP-NPs

ZrO-CLP-NPs ([ZrO]^2+^[CLP]^2–^ IOH-NPs) were obtained upon coprecipitation, relying on the low solubility of [ZrO]^2+^[CLP]^2–^. To this end, Na_2_(CLP) (25 mg, Aldrich, 95.7%) and the dye-modified nucleoside triphosphate DY-647-dUTP (1.2 mg, DY-647-dUTP, Dyomics, Germany) were dissolved in water (50 ml). The pH of this solution was adjusted to 7.0 upon the addition of diluted NaOH (140 µL, 0.5 M). Thereafter, an aqueous solution (5 ml) of ZrOCl_2_·8H_2_O (4.25 mg, Aldrich, 99%) was injected. After 2 min of intense stirring, IOH-NPs were separated via centrifugation (25,000 rpm, 15 min). To remove all the remaining salts, the colorless ZrO-CLP-NPs were resuspended and centrifuged in H_2_O three times. After redispersion, highly stable colloidal suspensions were obtained in water. The redispersed spherical NPs exhibited a mean diameter of 44 ± 11 nm (calculated by statistical evaluation of 100 particles on scanning electron microscopy (SEM) images). ZrO-CLP-NPs were characterized by 82 wt% CLP per NP. The release kinetics of CLP from ZrO-CLP-NPs were investigated elsewhere [22]. The hydrodynamic diameter was determined to be 73 (± 14) nm by dynamic light scattering (DLS). More details regarding the ZrO-CLP-NPs can be found in the previous data [21] and in Supporting Information, A. Characterization of the zirconyl clindamycinphosphate inorganic–organic hybrid nanoparticles ([ZrO]^2+^[CLP]^2−^ IOH-NPs, Figure S1-S6).

### Synthesis of superparamagnetic iron oxide NPs

Citrate-stabilized magnetite nanoparticles were synthesized in the first step based on a previously published protocol [43] with further modifications [43]. In brief, nitrogen was passed through 80 mL Millipore water for 10 min. Subsequently, iron(II) chloride tetrahydrate (2.722 g, Aldrich, ≥ 99%) and iron(III) chloride hexahydrate (7.4 g, Aldrich, 99%) were added and the reaction mixture was heated to 70 °C and stirred under reflux in a nitrogen atmosphere. After 30 min, an ammonium hydroxide solution (20 mL, Aldrich, ≥ 25% NH_3_ in water) was added and stirred for another 30 min at 70 °C, followed by the addition of citrate solution (2.5 g sodium tribasic dihydrate in 5 mL water, Aldrich, ≥ 99%). The temperature was increased to 90 °C and maintained for 60 min. The reaction mixture was then allowed to cool for 30 min. Magnetite nanoparticles were obtained by magnetic separation using DynaMag15 (Invitrogen, Carlsbad, United States). The particles were rinsed by redispersion in water using ultrasonication followed by magnetic separation. This process was repeated thrice, and the collected particles were dried overnight under vacuum. For macrophage loading, a freshly prepared suspension of SPIONs in H_2_O (30–40 mg/mL) was incubated for 20 min in a 4 °C sonifier water bath.

### Synthesis of MSNs

Synthesis was performed as published previously [44], with slight modifications (Supporting Information, B. Characterization of the mesoporous silica nanoparticles (MSN)). Briefly, CTAB (0.5 g), L-lysine (105 mg), and 210 mg of 2,2'-azobis(2-methyl-propiondiamidine)dihydrochloride (AIBA) were dissolved in 150 mL of water. Octane (98 mL) was added and the reaction mixture was degassed with nitrogen while stirring for 15 min. Subsequently, 105 μL of styrene and 5.18 mL of TEOS were added to the mixture, and the mixture was stirred at 61 °C under a slight nitrogen flow.

Subsequently, five spatulas of NaCl were added to the solution to promote particle agglomeration. After stirring for an additional 10 min, the mixture was centrifuged for 10 min at 18000xg and filtered. The precipitate was washed by dispersing it in water in an ultrasonic bath for ten minutes, followed by centrifugation for 15 min at 18000xg. This step was performed three times with water and once with absolute ethanol, and the particles were finally dried for 20 h at 60 °C. The particles were calcined at a heating rate of 1 °C∙ min^−1^ at 550 °C for 5 h, ground, and stored for further use. For characterization of MSNs see Supporting Information, Figure S7-S11.

### Characterization methods for MSNs

The TEM images were taken on a Tecnai G2 F20 TMP (Hillsboro, USA) microscope from FEI operated with 200 kV (Figure S7). The images were then processed using ImageJ software. For the preparation, the sample was dispersed in ethanol, the dispersion was dripped onto a copper grid (MicrotoNano, Harlem, Netherlands) and left to dry for several hours at room temperature. The MSN were measured using nitrogen (at 77 K) physisorption. The measurements were carried out on the gas sorption systems Autosorb-1 and Autosorb-3 from Quantachrome (Boynton Beach, USA) and the 3Flex from Micromeritics (Norcross, United States). The samples were outgassed in vacuum at 100 °C before the measurement. The data was evaluated using the ASiQwin 2.0 device software from Quantachrome and 3-Flex from Micromeritics GmbH. Surface areas were estimated by applying the Brunauer–Emmett–Teller (BET) equation. The pore size distribution was calculated using non-linear density-functional theory (NLDFT) and fitting of the Quantachrome Kernel “N2 at 77 K on silica for cylinder pores, NLDFT equilibrium model” to the experimental data. Values for total pore volumes were estimated by the single point method at p/p0 = 0.92 to exclude interparticular volume. Dynamic light scattering (DLS) measurements were performed on a Zetasizer Nano ZSP from Malvern Instruments (Worcestershire, UK) (Figure S8). The nanoparticles were redispersed in water by ultrasonification. Thermogravimetric analysis was conducted on a Simultaneous Thermal Analyzer 429 from NETZSCH (Selb, Germany) in air atmosphere (Figure S10). Measurements were carried out in a temperature range from 25 °C to 1000 °C with a heating rate of 5 K min^−1^. The measured data was evaluated using the instrument manufacturer's Proteus Thermal Analysis 4.3.1 software. IR spectra were recorded using an attenuated total reflectance (ATR) unit on a Tensor 27 Fourier transform IR spectrometer from Bruker (Billerica, Massachusetts, USA) in the range from 4000 cm^−1^ to 600 cm^−1^ (Figure S9). A measurement without a sample was carried out for the background measurement. The data was evaluated using the OPUS 5.0 software from Bruker.

### Loading MSNs with Dil, 4-OHT, Endoxifen and Raloxifene

MSNs were suspended in H_2_O (3 mg/mL) and sonified for 20 min in a 4 °C water bath. Stock solutions of 4-Hydroxytamoxifen (H6278, Sigma-Aldrich), Endoxifen (E8284, Sigma-Aldrich), and Raloxifene (R1402, Sigma-Aldrich) were prepared at a concentration of 20 mg/mL (Ethanol), 10 mg/mL (DMSO) and 20 mg/mL (DMSO), respectively. Suspensions of 3 mg/ml MSNs were made in PBS supplemented with the respective drugs at the following concentrations: 2 µg/mL 1,1'-Dioctadecyl-3,3,3',3'-Tetramethylindocarbocyanine Perchlorate ('DiI'; DiIC18(3), Invitrogen), 0.8 mg/mL 4-OHT, 0.8 mg/mL endoxifen or 0.8 mg/mL raloxifene. MSNs were incubated with the drugs overnight on a vertical rotor at 4 °C. The MSNs were centrifuged at 3500 rpm, washed four times with H_2_O and immediately used for loading of macrophages.

### Cells and cell culture

J774a.1, a murine macrophage line (DSMZ, Braunschweig, Germany), was cultured in DMEM (Gibco, Thermo Fisher Scientific) supplemented with 10% fetal bovine serum (FBS) (Sigma), 60 µg/mL penicillin and 100 µg/mL streptomycin. Bone marrow-derived macrophages (BMDMs) were obtained from the mice using a previously published protocol [45]. Briefly, bone marrow cells were seeded on bacterial culture plates with IMDM (Gibco, Thermo Fisher Scientific) supplemented with 10% FBS, 2 mM glutamine, 0.05 mM beta-mercaptoethanol, 60 µg/mL penicillin, and 100 µg/mL streptomycin. The cells were cultured with 20% L929 cell supernatant containing macrophage colony-stimulating factor (M-CSF) for the first four days and without M-CSF for the next three days. BMDMs were harvested on the 7th day for experiments. The Cre-luc reporter cells were cultured in DMEM supplemented with 10% FBS, 1 mM sodium pyruvate solution (Sigma-Aldrich), 10 mM HEPES, 1 × MEM, 0.05 mM beta-mercaptoethanol, 60 µg/mL penicillin, and 100 µg/mL streptomycin. All the cells were cultured at 37 °C in a humidified incubator with 5% CO_2_. To establish the M1 polarization, macrophages were stimulated with 50 ng/ml IFN-γ (Peprotech, Rocky Hill, NJ, USA) and 100 ng/ml LPS (no. L2880; Sigma-Aldrich, St. Louis, MO, USA) for 24 h. M2 polarization was established with 20 ng/ml IL-4 (214–14-50UG, PeproTech, USA) treatment for 24 h.

### Co-loading of macrophages with SPIONs and ZrO-CLP-NPs or MSNs

Macrophages were seeded at a density of 40,000 cells/cm^2^ in 12 well plates and cultured overnight prior to loading. For loading, the culture medium was removed from macrophages. Suspensions of drug-containing ZrO-CLP-NPs (40 µg/mL) or MSNs (80–200 µg/mL) in serum-free culture medium were added to the macrophages and incubated for 1.5 h or 2 h, respectively. Then, the supernatant was removed and cells were washed thrice with PBS to remove excess particles. Subsequently, SPIONs were suspended in serum-free culture medium at a concentration of 3–4 mg/mL and incubated with the cells for 1.5 h. Excess particles were removed by washing. All incubations of NPs and cells were carried out in a humidified 37 °C incubator with 5% CO_2_.

### Flow cytometry and cell sorting

For flow cytometry, cells were trypsined, washed and suspended in PBS supplemented with 2% of FBS. Flow cytometric analysis was performed using an LSR-II SORP and an LSR Fortessa instrument (both BD Biosciences). Specific cytometer settings are indicated in the section describing the respective method or within the figure legend. Cell sorting was performed using an Aria-II SORP (BD Biosciences). Data were processed using FlowJo v10.8.1 (BD Biosciences).

### Microscopy

Macrophages were stained with Cell Tracker Green CMFDA (C2925, Invitrogen) and Hoechst 33,258 (H-3569, Molecular Probes, Eugene, OR USA) following the manufacturer's instructions. Confocal sample analysis was performed using a Zeiss LSM 980 inverted laser-scanning microscope. Brightfield images and epifluorescence microscopy was perfomend using a Zeiss Observer.A1.

### AMF exposure of the NP-loaded cells

The coloaded macrophages were harvested and suspended in 80 µL of cell culture medium (2–2.5 × 10^5^ cells /80µL) in a microcentrifuge tube. The co-loaded cells were then exposed to AMF, with a constant field amplitude H_0_ (4.8 kA/m, 6, or 60 Oe) and a constant frequency *f* (779 kHz) using a magnetic field inductor (Hu5000 + , Himmel, Germany) for 20 min at room temperature. Following exposure to AMF, the cells were seeded and incubated in culture medium for up to 48 h.

### Cell death quantification and apoptotic assay

Cell death analysis was performed by incubation of macrophages with Nuclear Green DCS1 (ab138905, abcam) following the manufacturer's instructions. The frequency of Nuclear Green DCS1-labelled dead cells was determined by flow cytometry after gating for SSC^low^ and SSC^high^ populations (LSR Fortessa, bl525 channel with Ex 488 nm and Em BF 525/50 nm). The apoptotic activity of cells was determined by using the Annexin V-FITC Apoptosis Detection Kit ab14085 (Abcam). This dye fluorescently labels phosphatidylserine residues which are exposed at the outside of the cell membrane, which is an exclusive feature of apoptotic cells. The fluorescence was measured by flow cytometry (LSR-II SORP, FITC channel with Ex 488 nm and Em BF 525/50 nm). Raptinal, an apoptosis-inducing agent, was used at a concentration of 2 μM as control.

### Quantification of 4-OHT, reporter cell lysate preparation and luciferase assay

4-OHT in supernatants and cell lysates was quantified using the Cre-luc reporter cell line [46] generated from Rosa26-CreERT^2^ × ROSALUC mice [47]. In these cells, 4-OHT binds to the estrogen receptor moiety of the CreERT^2^ protein, thereby inducing its nuclear translocation and subsequent Cre-mediated recombination of the luciferase reporter gene. This results in a dose-dependent luciferase expression, with a sensitivity of 2 ng/ml 4-OHT (Figure S15). Cre-luc reporter cells were seeded in 24-well plates (6 × 10^4^ cells/well). Transwell inserts (Corning, 3470) were added to the reporter cell plates, followed by the addition of 4-OHT NP-loaded macrophages to the inside compartment. Cells were co-cultured for 24 h or 48 h. Alternatively, the reporter cells were seeded at a density of 3 × 10^4^ cells/well in 48-well plates and incubated for 24 h with the culture supernatant that had been harvested 24 h or 48 h after loading of macrophages. After transwell co-culture or exposure to the culture supernatants, reporter activity was determined. To this end, Cre-luc cells were washed with PBS and incubated with adjusted amounts of reporter lysis buffer (RLB, E4030 Promega) at −70 °C for at least 60 min. A single-tube luminometer (Lumat LB 9507, Berthold Technologies) was used to assess the luciferase activity of the lysates [48]. The relative light units (RLUs) obtained after 10 s were related to protein content (RLU/mg).

### RNA isolation and RT-qPCR

Total RNA was prepared from 3–4 × 10^5^ cells using the RNeasy Mini Kit (Qiagen, Germany) following the manufacturer's instructions. The RNA samples were eluted in 30 μl RNase-free H_2_O. Quality and quantity of the isolated RNA samples were assessed using the NanoDrop ND-1000 spectrophotometer. Reverse transcription was carried out using the RevertAid RT Kit (K1691, ThermoScientific) and Oligo-dT primers (Qiagen) according to the manufacturer's instructions. Briefly, a mixture of Oligo-dT primers and eluted RNA was denatured at 65 °C for 5 min, followed by 5 min incubation on ice and transferred to the reaction mastermix (RT buffer, dNTPs, RI and RT) for reverse transcription at 45 °C for 1 h. 1:10 diluted cDNAs were used as a template for amplification with specific primers for coding sequences of *Tnf*a (Tnfa-F: 5’-GGAACTGGCAGAAGAGGCACT-3’, Tnfa-R: 5’-GCAGGAATGAGAAGAGGCTGAGAC-3’) and *Mrc1* (Mrc1-F: 5’-TAGATGGAGGGTGCGGTACA-3’, Mrc1-R: 5’-TCCACGCAGTCTGTTCTGAC-3’). For real-time PCR the following components were mixed: 10 μl 2 × EvaGreen (Biorad) RT-PCR mix, 1 μl forward primer (10 pmol), 1 μl reverse primer (10 pmol), 8 μl cDNA mix. Real-time PCR was performed on a LightCycler 480 II apparatus (Roche). The housekeeping gene beta-actin (*Actb*) (Actinb-F: 5′-TGGAATCCTGTGGCATCCATGAAA-3’, Actinb-R: 5′-TAAAACGCAGCTCAGTAACAGTCCG-3') was used for normalization. Reactions were performed in triplicates.

### Bacterial growth inhibition assay

*Staphylococcus aureus* strain SH1000 was grown to mid-log phase at 37 °C with shaking (150 rpm) in brain heart infusion (BHI) medium [22]. The bacterial cells were seeded in a 96-well plate. Serial dilutions of the treatments of interest were added to the seeded bacterial cells: DMEM control, supernatant from loaded J774a.1 cells (after centrifugation at 5000 rpm for 5 min at 4 °C), and CLP at a starting concentration of 5 µg/mL. Bacteria were incubated at 37 °C for 18–20 h, after which OD595 was measured to determine bacterial growth.

### Imaging and statistical analysis

Microscopy images were analyzed using ImageJ (NIH, Bethesda, Maryland, USA) and the built-in plug-in software. All data analyses were performed using GraphPad Prism v10.1.1 (Graph Pad Software, La Jolla, CA, USA). The results are presented as mean ± SEM from triplicate samples or from the numbers indicated in the figure legends. Statistical significance was tested using unpaired Student’s t-test. P-values less than 0.05 were considered to be statistically significant.

## Supplementary Information

Below is the link to the electronic supplementary material.

Supplementary file1 (PDF 3306 KB)

## Data Availability

The data are available on request.

## References

[1] Mitchell MJ, Billingsley MM, Haley RM, Wechsler ME, Peppas NA, Langer R. Engineering precision nanoparticles for drug delivery. Nat Rev Drug Discovery. 2021;20:101–24. 33277608 10.1038/s41573-020-0090-8 PMC7717100

[2] Priya S, Desai VM, Singhvi G. Surface Modification of Lipid-Based Nanocarriers: A Potential Approach to Enhance Targeted Drug Delivery. ACS Omega. 2023;8:74–86. 36643539 10.1021/acsomega.2c05976 PMC9835629

[3] Sanita G, Carrese B, Lamberti A. Nanoparticle Surface Functionalization: How to Improve Biocompatibility and Cellular Internalization. Front Mol Biosci. 2020;7: 587012. 33324678 10.3389/fmolb.2020.587012 PMC7726445

[4] Ho D, Sun X, Sun S. Monodisperse magnetic nanoparticles for theranostic applications. Acc Chem Res. 2011;44:875–82. 21661754 10.1021/ar200090c PMC3184307

[5] Dai Q, Wilhelm S, Ding D, Syed AM, Sindhwani S, Zhang Y, Chen YY, MacMillan P, Chan WCW. Quantifying the Ligand-Coated Nanoparticle Delivery to Cancer Cells in Solid Tumors. ACS Nano. 2018;12:8423–35. 30016073 10.1021/acsnano.8b03900

[6] Wilhelm S, Tavares AJ, Dai Q, Ohta S, Audet J, Dvorak HF, Chan WCW. Analysis of nanoparticle delivery to tumours. Nat Rev Mater. 2016;1:1–2.

[7] Xuan M, Shao J, Li J. Cell membrane-covered nanoparticles as biomaterials. Natl Sci Rev. 2019;6:551–61. 34691904 10.1093/nsr/nwz037 PMC8291551

[8] Yan H, Shao D, Lao YH, Li M, Hu H, Leong KW. Engineering Cell Membrane-Based Nanotherapeutics to Target Inflammation. Adv Sci (Weinh). 2019;6:1900605. 31406672 10.1002/advs.201900605 PMC6685500

[9] Wroblewska A, Szczygiel A, Szermer-Olearnik B, Pajtasz-Piasecka E. Macrophages as Promising Carriers for Nanoparticle Delivery in Anticancer Therapy. Int J Nanomed. 2023;18:4521–39. 10.2147/IJN.S421173 PMC10422973 37576466

[10] Zhang W, Taheri-Ledari R, Ganjali F, Mirmohammadi SS, Qazi FS, Saeidirad M, KashtiAray A, Zarei-Shokat S, Tian Y, Maleki A. Effects of morphology and size of nanoscale drug carriers on cellular uptake and internalization process: a review. RSC Adv. 2022;13:80–114. 36605676 10.1039/d2ra06888e PMC9764328

[11] Garapaty A, Champion JA. Tunable particles alter macrophage uptake based on combinatorial effects of physical properties. Bioeng Transl Med. 2017;2:92–101. 29313025 10.1002/btm2.10047 PMC5689517

[12] Pang L, Qin J, Han L, Zhao W, Liang J, Xie Z, Yang P, Wang J. Exploiting macrophages as targeted carrier to guide nanoparticles into glioma. Oncotarget. 2016;7:37081–91. 27213597 10.18632/oncotarget.9464 PMC5095060

[13] Si J, Shao S, Shen Y, Wang K. Macrophages as Active Nanocarriers for Targeted Early and Adjuvant Cancer Chemotherapy. Small. 2016;12:5108–19. 27560388 10.1002/smll.201601282

[14] Chen W, Schilperoort M, Cao Y, Shi J, Tabas I, Tao W. Macrophage-targeted nanomedicine for the diagnosis and treatment of atherosclerosis. Nat Rev Cardiol. 2022;19:228–49. 34759324 10.1038/s41569-021-00629-x PMC8580169

[15] Liang T, Zhang R, Liu X, Ding Q, Wu S, Li C, Lin Y, Ye Y, Zhong Z, Zhou M. Recent Advances in Macrophage-Mediated Drug Delivery Systems. Int J Nanomed. 2021;16:2703–14. 10.2147/IJN.S298159 PMC8039204 33854316

[16] Watermann A, Brieger J. Mesoporous Silica Nanoparticles as Drug Delivery Vehicles in Cancer. Nanomaterials (Basel). 2017;7:189. 28737672 10.3390/nano7070189 PMC5535255

[17] Bharti C, Nagaich U, Pal AK, Gulati N. Mesoporous silica nanoparticles in target drug delivery system: A review. Int J Pharm Investig. 2015;5:124–33. 26258053 10.4103/2230-973X.160844 PMC4522861

[18] Heck JG, Napp J, Simonato S, Mollmer J, Lange M, Reichardt HM, Staudt R, Alves F, Feldmann C. Multifunctional phosphate-based inorganic-organic hybrid nanoparticles. J Am Chem Soc. 2015;137:7329–36. 26018463 10.1021/jacs.5b01172

[19] Ischyropoulou M, Sabljo K, Schneider L, Niemeyer CM, Napp J, Feldmann C, Alves F. High-Load Gemcitabine Inorganic-Organic Hybrid Nanoparticles as an Image-Guided Tumor-Selective Drug-Delivery System to Treat Pancreatic Cancer. Adv Mater. 2023;35: e2305151. 37587542 10.1002/adma.202305151

[20] Montes-Cobos E, Ring S, Fischer HJ, Heck J, Strauss J, Schwaninger M, Reichardt SD, Feldmann C, Luhder F, Reichardt HM. Targeted delivery of glucocorticoids to macrophages in a mouse model of multiple sclerosis using inorganic-organic hybrid nanoparticles. J Control Release. 2017;245:157–69. 27919626 10.1016/j.jconrel.2016.12.003

[21] Napp J, Markus MA, Heck JG, Dullin C, Mobius W, Gorpas D, Feldmann C, Alves F. Therapeutic Fluorescent Hybrid Nanoparticles for Traceable Delivery of Glucocorticoids to Inflammatory Sites. Theranostics. 2018;8:6367–83. 30613305 10.7150/thno.28324 PMC6299685

[22] Heck JG, Rox K, Lunsdorf H, Luckerath T, Klaassen N, Medina E, Goldmann O, Feldmann C. Zirconyl Clindamycinphosphate Antibiotic Nanocarriers for Targeting Intracellular Persisting Staphylococcus aureus. ACS Omega. 2018;3:8589–94. 31458988 10.1021/acsomega.8b00637 PMC6644946

[23] Andreu I, Natividad E, Solozabal L, Roubeau O. Nano-objects for addressing the control of nanoparticle arrangement and performance in magnetic hyperthermia. ACS Nano. 2015;9:1408–19. 25658023 10.1021/nn505781f

[24] Dulinska-Litewka J, Lazarczyk A, Halubiec P, Szafranski O, Karnas K, Karewicz A. Superparamagnetic Iron Oxide Nanoparticles-Current and Prospective Medical Applications. Materials (Basel). 2019;12:617. 30791358 10.3390/ma12040617 PMC6416629

[25] MashhadiMalekzadeh A, Ramazani A, TabatabaeiRezaei SJ, Niknejad H. Design and construction of multifunctional hyperbranched polymers coated magnetite nanoparticles for both targeting magnetic resonance imaging and cancer therapy. J Colloid Interface Sci. 2017;490:64–73. 27870961 10.1016/j.jcis.2016.11.014

[26] Jarrett BR, Frendo M, Vogan J, Louie AY. Size-controlled synthesis of dextran sulfate coated iron oxide nanoparticles for magnetic resonance imaging. Nanotechnology. 2007;18: 035603. 19636126 10.1088/0957-4484/18/3/035603

[27] Janko C, Ratschker T, Nguyen K, Zschiesche L, Tietze R, Lyer S, Alexiou C. Functionalized Superparamagnetic Iron Oxide Nanoparticles (SPIONs) as Platform for the Targeted Multimodal Tumor Therapy. Front Oncol. 2019;9:59. 30815389 10.3389/fonc.2019.00059 PMC6382019

[28] Naud C, Thebault C, Carriere M, Hou Y, Morel R, Berger F, Dieny B, Joisten H. Cancer treatment by magneto-mechanical effect of particles, a review. Nanoscale Adv. 2020;2:3632–55. 36132753 10.1039/d0na00187b PMC9419242

[29] Kalber TL, Ordidge KL, Southern P, Loebinger MR, Kyrtatos PG, Pankhurst QA, Lythgoe MF, Janes SM. Hyperthermia treatment of tumors by mesenchymal stem cell-delivered superparamagnetic iron oxide nanoparticles. Int J Nanomed. 2016;11:1973–83. 10.2147/IJN.S94255 PMC4869665 27274229

[30] Vangijzegem T, Lecomte V, Ternad I, Van Leuven L, Muller RN, Stanicki D, Laurent S. Superparamagnetic Iron Oxide Nanoparticles (SPION): From Fundamentals to State-of-the-Art Innovative Applications for Cancer Therapy. Pharmaceutics. 2023;15:236. 36678868 10.3390/pharmaceutics15010236 PMC9861355

[31] Pucci C, Degl’Innocenti A, BelenliGumus M, Ciofani G. Superparamagnetic iron oxide nanoparticles for magnetic hyperthermia: recent advancements, molecular effects, and future directions in the omics era. Biomater Sci. 2022;10:2103–21. 35316317 10.1039/d1bm01963e

[32] Ullah S, Seidel K, Turkkan S, Warwas DP, Dubich T, Rohde M, Hauser H, Behrens P, Kirschning A, Koster M, Wirth D. Macrophage entrapped silica coated superparamagnetic iron oxide particles for controlled drug release in a 3D cancer model. J Control Release. 2019;294:327–36. 30586597 10.1016/j.jconrel.2018.12.040

[33] Azeh I, Gerber J, Wellmer A, Wellhausen M, Koenig B, Eiffert H, Nau R. Protein synthesis inhibiting clindamycin improves outcome in a mouse model of Staphylococcus aureus sepsis compared with the cell wall active ceftriaxone. Crit Care Med. 2002;30:1560–4. 12130979 10.1097/00003246-200207000-00027

[34] Jordan VC. New insights into the metabolism of tamoxifen and its role in the treatment and prevention of breast cancer. Steroids. 2007;72:829–42. 17765940 10.1016/j.steroids.2007.07.009 PMC2740485

[35] Mantovani A, Allavena P, Marchesi F, Garlanda C. Macrophages as tools and targets in cancer therapy. Nat Rev Drug Discovery. 2022;21:799–820. 35974096 10.1038/s41573-022-00520-5 PMC9380983

[36] Chen S, Saeed A, Liu Q, Jiang Q, Xu H, Xiao GG, Rao L, Duo Y. Macrophages in immunoregulation and therapeutics. Signal Transduct Target Ther. 2023;8:207. 37211559 10.1038/s41392-023-01452-1 PMC10200802

[37] Mosser DM, Hamidzadeh K, Goncalves R. Macrophages and the maintenance of homeostasis. Cell Mol Immunol. 2021;18:579–87. 32934339 10.1038/s41423-020-00541-3 PMC7491045

[38] Neumeier BL, Khorenko M, Alves F, Goldmann O, Napp J, Schepers U, Reichardt HM, Feldmann C. Fluorescent Inorganic-Organic Hybrid Nanoparticles. ChemNanoMat. 2019;5:24–45.

[39] Ackermann M, RafieiHashtchin A, Manstein F, Carvalho Oliveira M, Kempf H, Zweigerdt R, Lachmann N. Continuous human iPSC-macrophage mass production by suspension culture in stirred tank bioreactors. Nat Protoc. 2022;17:513–39. 35039668 10.1038/s41596-021-00654-7 PMC7612500

[40] Ackermann M, Kempf H, Hetzel M, Hesse C, Hashtchin AR, Brinkert K, Schott JW, Haake K, Kuhnel MP, Glage S, Figueiredo C, Jonigk D, Sewald K, Schambach A, Wronski S, Moritz T, Martin U, Zweigerdt R, Munder A, Lachmann N. Bioreactor-based mass production of human iPSC-derived macrophages enables immunotherapies against bacterial airway infections. Nat Commun. 2018;9:5088. 30504915 10.1038/s41467-018-07570-7 PMC6269475

[41] Na YR, Kim SW, Seok SH. A new era of macrophage-based cell therapy. Exp Mol Med. 2023;55:1945–54. 37653035 10.1038/s12276-023-01068-z PMC10545778

[42] Guo Q, Qian ZM. Macrophage based drug delivery: Key challenges and strategies. Bioact Mater. 2024;38:55–72. 38699242 10.1016/j.bioactmat.2024.04.004 PMC11061709

[43] Hagemann V, Finck L, Herrmann T, Menzel H, Ehlert N. Core-Shell-Nanoparticles with Superparamagnetic Properties for Novel Applications as Biomaterials. Curr Dir Biomed Eng. 2023;9:666–9.

[44] Nandiyanto ABD, Kim S-G, Iskandar F, Okuyama K. Synthesis of spherical mesoporous silica nanoparticles with nanometer-size controllable pores and outer diameters. Microporous Mesoporous Mater. 2009;120:447–53.

[45] Toda G, Yamauchi T, Kadowaki T, Ueki K. Preparation and culture of bone marrow-derived macrophages from mice for functional analysis. STAR Protoc. 2021;2: 100246. 33458708 10.1016/j.xpro.2020.100246 PMC7797923

[46] Desai O, Köster M, Lachmann N, Hauser H, Poortinga A, Wirth D (in press). Ultrasound-mediated drug release from antibubble-loaded macrophages in vivo. J Control Release 10.1016/j.jconrel.2024.12.007 39653149

[47] Sandhu U, Cebula M, Behme S, Riemer P, Wodarczyk C, Metzger D, Reimann J, Schirmbeck R, Hauser H, Wirth D. Strict control of transgene expression in a mouse model for sensitive biological applications based on RMCE compatible ES cells. Nucleic Acids Res. 2011;39: e1. 20935052 10.1093/nar/gkq868 PMC3017619

[48] Godecke N, Riedel J, Herrmann S, Behme S, Rand U, Kubsch T, Cicin-Sain L, Hauser H, Koster M, Wirth D. Synthetic rewiring and boosting type I interferon responses for visualization and counteracting viral infections. Nucleic Acids Res. 2020;48:11799–811. 33137201 10.1093/nar/gkaa961 PMC7672444

