# Supplementary material for: Macrophages co-loaded with drug-associated and superparamagnetic nanoparticles for triggered drug release by alternating magnetic fields
Source: Drug Deliv Transl Res. 2025 Jan 13;15(8):2779–93. doi: 10.1007/s13346-024-01774-9 (PMC12213844; doi:10.1007/s13346-024-01774-9)
Supplement: Supplementary file 1 — Supplementary file1 (PDF 3306 KB) [file 13346_2024_1774_MOESM1_ESM.pdf]

Supporting Information

Double loading of macrophages with drug-associated and superparamagnetic nanoparticles allows specific cargo release by alternating magnetic fields

Omkar Desai[1]*, Sandhya Kumar Kanaujia[1]*, Mario Köster[1]*, Sami Ullah[1+], Sushobhan Sarker[1], Valentin Hagemann[2], Mosaieb Habib[2], Nicole Klaassen[3], Silke Notter[3], Claus Feldmann[3], Nina Ehlert[2], Hansjörg Hauser[1,4], Dagmar Wirth[1,5]

Overview

A. **Characterization of the zirconyl clindamycinphosphate inorganic-organic hybrid nanoparticles ($[ZrO]^{2+}[CLP]^{2-}$ IOH-NPs)**

B. **Characterization of the mesoporous silica nanoparticles (MSN)**

C. **Supplementary experiments**

C1: Quantification of NP mass taken up by macrophages

C2: qRT-PCR analysis of M1/M2 markers upon loading ZrO-CLP-NPs

C3: Quantification of type I/III IFN production by loaded macrophages

C4: Quantification of 4-OHT based on Cre-Luc reporter cells

D. **References**

A. Characterization of the zirconyl clindamycinphosphate inorganic-organic hybrid nanoparticles ($[ZrO]^{2+}[CLP]^{2-}$ IOH-NPs)

According to our concept of inorganic-organic hybrid nanoparticles (IOH-NPs) [S1], these IOH-NPs consist of a phosphate- or carboxylate-functionalized drug anion and a specific inorganic cation to make the combination of drug anion and inorganic cation insoluble in water (Figure S1). The general aims of IOH-NPs relate to a drug load per nanoparticle as high as possible as well as to a simple synthesis in water. Here, we use the antibiotic agent clindamycinphosphate, $[CLP]^{2-}$, as the drug anion (Figure S1) and the inorganic zirconyl anion, $[ZrO]^{2+}$, to obtain $[ZrO]^{2+}[CLP]^{2-}$ IOH-NPs as poorly soluble compound in water. The resulting saline $[ZrO]^{2+}[CLP]^{2-}$ IOH-NPs contain an unprecedented high drug load of 82 % of the total nanoparticle mass (remaining 18 % relate to inorganic cation; Figure S1). Synthesis and characterization of the $[ZrO]^{2+}[CLP]^{2-}$ IOH-NPs were previously reported in reference [S2].

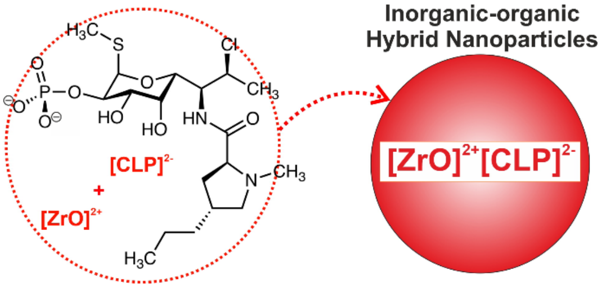

Figure S1: Scheme illustrating the water-based synthesis of $[ZrO]^{2+}[CLP]^{2-}$ inorganic-organic hybrid nanoparticles (IOH-NPs) (modified reproduction from [S2]).

Scanning electron microscopy (SEM) (Figure S2) indicates the presence of spherical nanoparticles with a mean diameter of 44±11 nm (calculated by statistical evaluation of 100 particles).

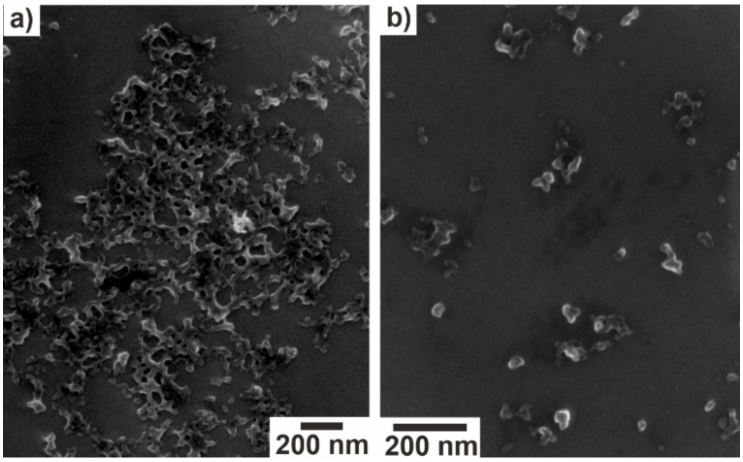

Figure S2: SEM images of $[ZrO]^{2+}[CLP]^{2-}$ IOH-NPs with overview image (a) and single nanoparticles (b) (modified reproduction from [S2]).

Dynamic light scattering (DLS) was used to determine the particle size of the $[ZrO]^{2+}[CLP]^{2-}$ IOH-NPs in aqueous suspension. Thus, the hydrodynamic diameter results in 73±14 nm (Figure S3a). As expected, mean diameter and size distribution obtained by DLS are larger as compared to the values obtained by SEM due to surface-adsorbed water. Because of the high polarity of water and the presence of strong hydrogen bonding, an expanded rigid solvent shell occurred. A photo of an aqueous suspension with $[ZrO]^{2+}[CLP]^{2-}$ IOH-NPs qualitatively shows the good colloidal stability (Figure S3b).

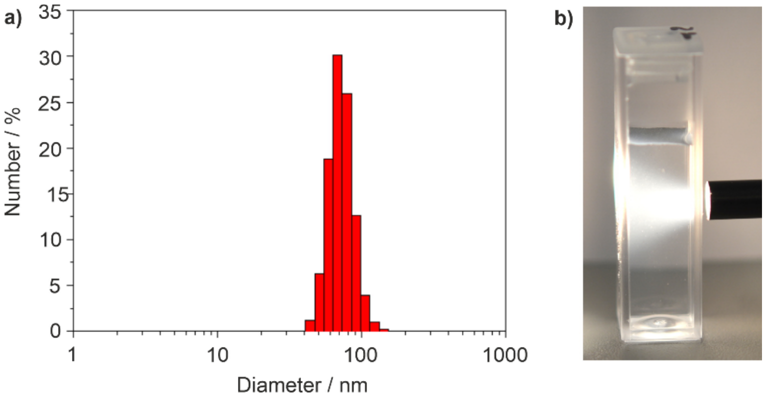

Figure S3: DLS analysis of the as-prepared $[ZrO]^{2+}[CLP]^{2-}$ IOH-NPs (in water) (a) with photo of aqueous suspension (b) (modified reproduction from [S2]).

Fourier-transform infrared spectroscopy (FT-IR), elemental analysis (EA) and thermogravimetry (TG) validate the chemical composition of the $[ZrO]^{2+}[CLP]^{2-}$ IOH-NPs. First of all, FT-IR spectra evidence the presence of $[CLP]^{2-}$ in the $[ZrO]^{2+}[CLP]^{2-}$ IOH-NPs (Figure S4). All characteristic vibrations of clindamycinphosphate are very comparable for the $[ZrO]^{2+}[CLP]^{2-}$ IOH-NPs and $Na_2(CLP)$ as the starting material (ν(O−H) at 3600-3200 cm^{-1}, ν(C–H): 3000-2800 cm^{-1}, ν(C=O): 1750-1500 cm^{-1}, $\nu(PO_4)$: 1250-1000 cm^{-1}, *fingerprint area*: 1500-1250 and 1000-500 cm^{-1}). The strong vibration ν(O−H), finally, indicates the presence of H_2O.

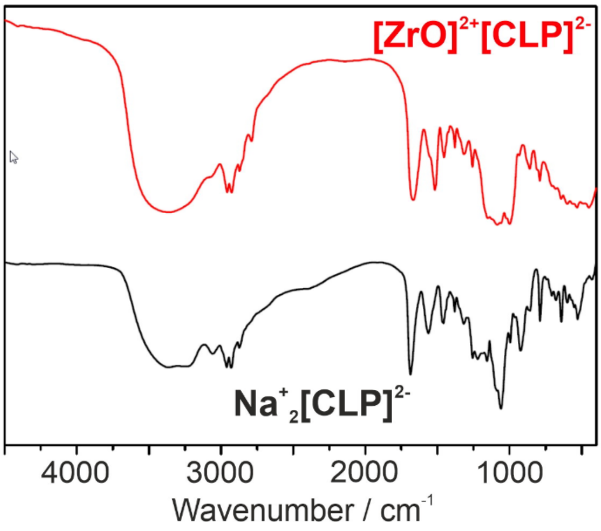

Figure S4: FT-IR spectra of $[ZrO]^{2+}[CLP]^{2-}$ IOH-NPs (with $Na_2(CLP)$ as a reference) (modified reproduction from [S2]).

To quantify the chemical composition of the $[ZrO]^{2+}[CLP]^{2-}$ IOH-NPs, EA was performed and resulted in an element composition of 33.0 wt-% C, 5.6 wt-% H, 5.6 wt-% N, 4.6 wt-% S, which is well in agreement with the calculated data (C: 35.0, H: 4.6, N: 5.3, S: 5.3 wt-%). Furthermore, total organic combustion/thermogravimetry (TG) was performed to verify the organic content via thermal combustion (Figure S5). Here, $[ZrO]^{2+}[CLP]^{2-}$ shows a more-or-less continuous decomposition in a temperature range of 100-1000 °C with a total mass loss of 66% (samples pre-dried at 100 °C) due to total combustion of the $[CLP]^{2-}$ anion. This value matches very well with the calculated mass loss of 68%. The remnant of the thermal decomposition was identified via X-ray powder diffraction as a mixture of ZrO_2 and $Zr_3(PO_4)_4$. In sum, the total organics combustion of $[ZrO]^{2+}[CLP]^{2-}$ can be rationalized based on the following reaction:

$$4[ZrO]^{2+}[C_{18}H_{33}PSClN_2O_8]^{2-} + 99O_2 \rightarrow ZrO_2 + Zr_3(PO_4)_4 + 72CO_2\uparrow + 4N_2\uparrow + 4SO_2\uparrow + 4HCl\uparrow + 64H_2O\uparrow$$

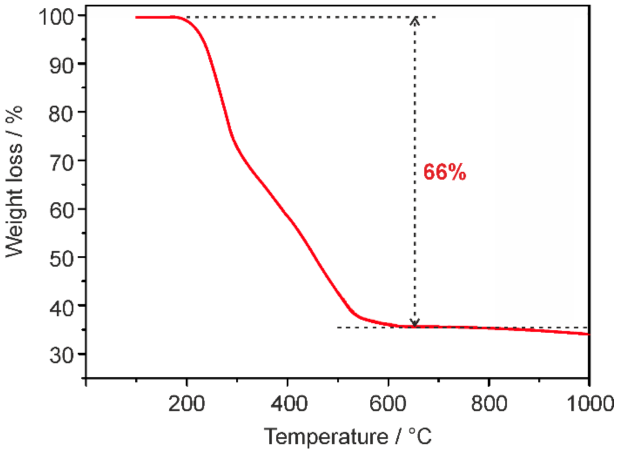

Figure S5: TG of the as-prepared $[ZrO]^{2+}[CLP]^{2-}$ IOH-NPs (modified reproduction from [S2]).

To allow monitoring of the IOH-NPs via fluorescence imaging, they were labelled by addition of low amounts (0.005 mol-%) of the near-infrared emitting dye-modified nucleoside triphosphate DY-647-dUTP ($[DUT]^{2-}$, Dyomics, Germany). After the synthesis, the presence of the dye in the resulting $[ZrO]^{2+}[(CLP)_{0.995}(DUT)_{0.005}]^{2-}$ IOH-NPs is already validated by the slightly bluish color of the IOH-NPs as well as by the intense deep red to infrared fluorescence (Figure S6). Excitation and emission spectra of fluorescence-marked $[ZrO]^{2+}[(CLP)_{0.995}(DUT)_{0.005}]^{2-}$ IOH-NPs are shown in Figure S6. Accordingly, the excitation can be performed at 500-700 nm with λ_{max} = 655 nm. Emission occurs at 630-780 nm with λ_{max} = 675 nm. The emission of the $[ZrO]^{2+}[(CLP)_{0.995}(DUT)_{0.005}]^{2-}$ IOH-NPs - as expected - is identical to pure Dyomics-647 uridine triphosphate dye.

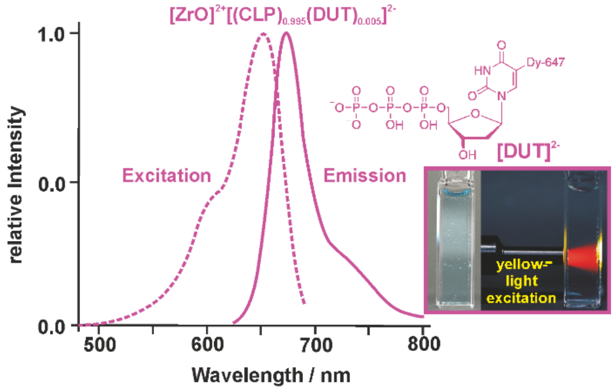

Figure S6: Excitation and emission spectra of $[ZrO]^{2+}[(CLP)_{0.995}(DUT)_{0.005}]^{2-}$ IOH-NPs (modified reproduction from [S2]).

B. Characterization of the mesoporous silica nanoparticles (MSN)

The mesoporous silica nanoparticles (MSN) were synthesized according to Nandiyanto et al. (2009). Transmission electron microscopy (TEM) was carried out to determine the size and morphology of the particles. As depicted in Figure S7 the spherical as-synthesized nanoparticles have a size of 75 nm ± 7 nm, and the calcined nanoparticles of 73 nm ± 6 nm (average of 20 particles), which is slightly different as expected by literature (app. 50 nm) probably caused by higher reaction volume. Both show a porous system in the nanometer range. According to literature the heat treatment of calcination does not lead to swelling or shrinkage of the particles (Nandiyanto et al., 2009).

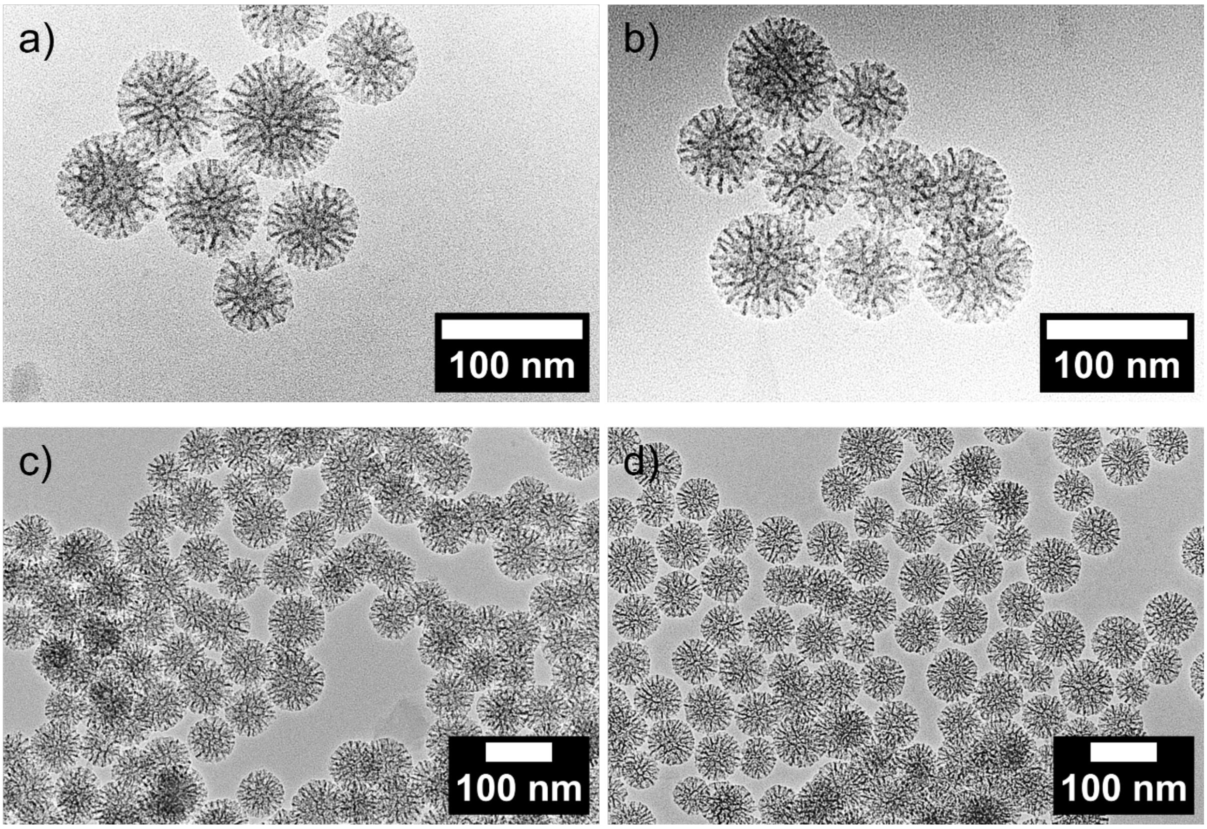

Figure S7: Transmission electron microscopy images of the synthesized MSN. a) & c) before and b) & d) after calcination. Both particles show a spherical shape with a nanoporous structure and an average pore size of 75 nm ± 7 nm before and 73 nm ± 6 nm after calcination.

The particle size distribution, as determined by dynamic light scattering (DLS) (Fig. S8), is lying in the same range. Before calcination, the mean hydrodynamic diameter is 83 nm ± 4 nm (MSN_PS), and after calcination 78 nm ± 9 nm (MSN_calc). The polydispersity index (PDI) for both samples was approximately 0.3 and thus they could be classified as moderately polydisperse. The slightly increased particle sizes compared to the TEM measurements can be

attributed to the presence of a hydrate shell around the particles and possible agglomerates or aggregates, which are also detected in the DLS measurement.

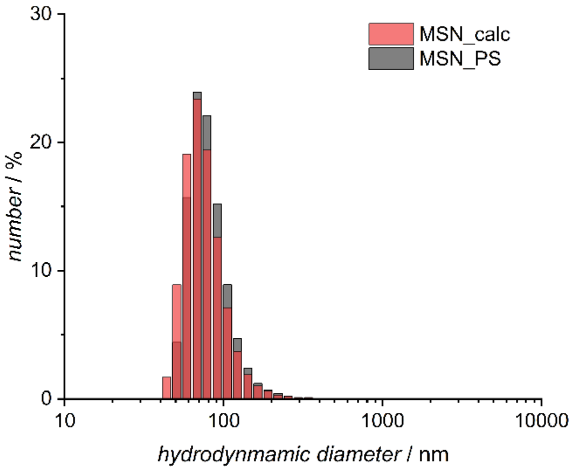

Figure S8: Number-weighted size distribution of MSN_PS and MSN_calc using DLS. The size distribution of the two samples is in a similar range. The mean hydrodynamic diameters are 83 nm ± 4 nm (MSN_PS) and 78 nm ± 9 nm (MSN_calc).

Figure S9 demonstrates the recorded IR spectra of the untreated and calcined MSN similar to those in literature (Nandiyanto et al., 2009). The characteristic vibrational bands of silica can be seen in both spectra. The most intense band at 1060 cm^{-1} is caused by the (Si-O-Si) valence vibration. Furthermore, the bands at wavenumbers of 970 cm^{-1}, 800 cm^{-1}, and 460 cm^{-1} can be assigned to the (Si-OH) valence vibration, (Si-O), and (O-Si-O) deformation vibration. The bands at 3400 cm^{-1} and 1650 cm^{-1} are caused by surface silanol (Si-OH) groups or adsorbed water on the surfaces of the particles. The two spectra differ mainly in the bands at 2925 cm^{-1}, 2855 cm^{-1}, and 1480 cm^{-1}, the first two resulting from the (C-H) valence vibrations and the last from the (C-H) deformation vibration. These bands are caused by the templates polystyrene and cetyltrimethylammonium bromide (CTAB). After calcination, these bands are no longer present due to the removal of the organic templates.

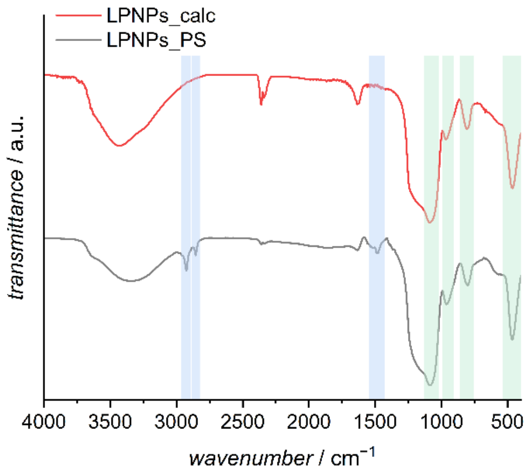

Figure S9: IR spectra of the MSN before (MSN_PS) and after calcination (MSN_calc). Both samples show the characteristic bands of silica (green bars). In the MSN_PS, the bands caused by alkyl groups of CTAB and polystyrene (blue bars) are present, in the MSN_calc they vanish due to the removal of the templates.

To determine the organic content in the mesoporous particles caused by CTAB and polystyrene, thermogravimetric measurements were conducted, which are shown in Figure S10. Both measurements initially show a 2% mass loss up to 150 °C, which is caused by the removal of the adsorbed solvent. A further mass loss occurs in the range from 150 °C to 600 °C. This second step can mainly be attributed to the combustion of the organic components, although a small proportion is also caused by the dehydroxylation of the silica surface due to further condensation of the silanol groups. The exact determination of the mass loss due to the dehydroxylation of the surface of the MSN was not possible within the scope of these measurements. For the MSN_PS, the second step with a mass loss of 24% is significantly higher than for the MSN_calc with 1%. These were already heated to 550 °C during the calcination which led to the removal of the organic components.

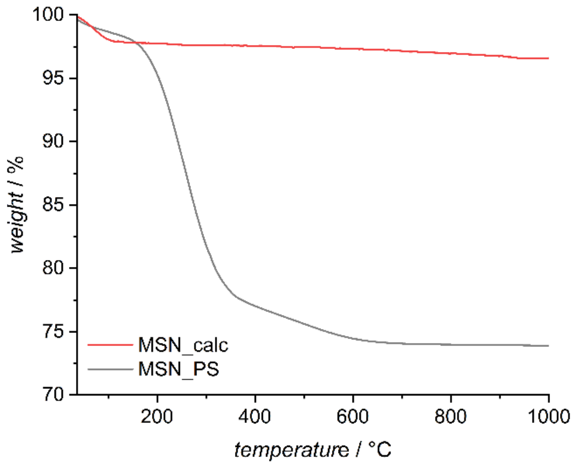

Figure S10: Thermogravimetric analysis of the MSN_PS and MSN_calc. In the range from 150 °C to 600 °C, the mass loss is mainly caused by the combustion of polystyrene and CTAB. With 24%, the mass loss in this temperature range is significantly higher for the MSN_PS than for the MSN_calc.

Physisorption with nitrogen as an adsorptive was used to investigate the pore system of the MSN. The recorded isotherms can be seen in Figure S11a. The steeper increase in the isotherm of MSN_calc at low relative pressures indicates that this sample has a larger specific surface area compared to MSN_PS. This is also confirmed by the specific surface areas determined using the Brunauer-Emmett-Teller (BET) method. The BET surface area of the MSN increases from 360 m^2 g^{-1} to 810 m^2 g^{-1} after calcination, which corresponds to a more than twofold increase. This increase is evoked by the removal of the SDAs from the pores of the particles, which makes the pore system accessible. This effect is also demonstrated by the determined pore volume, it increases from 0.8 cm^3 g^{-1} to 1.2 cm^3 g^{-1}. The DFT pore size distribution of MSN_PS and MSN_calc is shown in Figure S11b. The maxima of the pore size distribution differ between the two samples. The average pore size is 7 nm before calcination. After calcination, two maxima at 3.2 nm and around 8 nm appear. A possible explanation for this is that the template polystyrene and CTAB clog the smaller 3.2 nm pores, but not the larger 8 nm pores. After calcination, the smaller pores become accessible. Thus, after the removal of the SDAs, the MSN have two pores of different sizes.

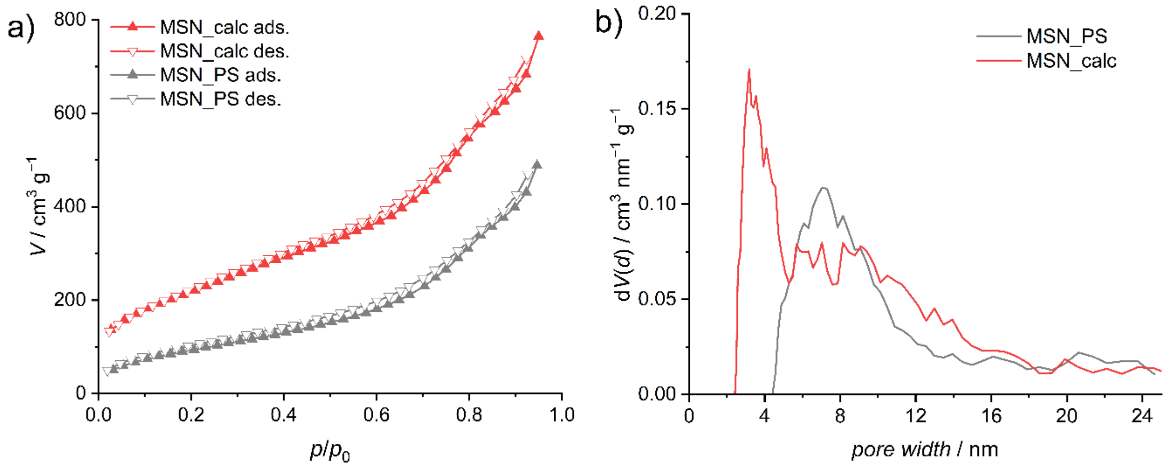

Figure S11: N_2-Physisorption isotherms (a) and DFT pore size distributions (b) of the nanoporous silica nanoparticles before (MSN_PS) and after (MSN_calc) calcination. The BET surface area of the MSN increases from 360 $m^2\ g^{-1}$ to 810 $m^2\ g^{-1}$ after removal of the SDAs. The average pore size of the MSN_PS is 7 nm. For the MSN_calc, two maxima can be recognized at 3.2 nm and 8 nm.

Except for styrene, the chemicals listed below were used without further purification. 2,2'-Azobis(2-methyl-propiondiamidine) dihydrochloride (AIBA, 97%), L-lysine (≥98%), styrene (98%), tetraethyl orthosilicate (TEOS, ≥99%), and cetyl trimethyl ammonium bromide (CTAB, 98%) were purchased from Sigma-Aldrich Corporation (München, Germany). Octane (98%) was delivered from ABCR GmbH & Co. KG (Karlsruhe, Germany). Absolute ethanol was purchased from Merck (Darmstadt, Germany). Styrene was shaken out three times with a 2.5 M sodium hydroxide solution. For 10 mL of styrene, 5 mL of sodium hydroxide solution was used for each step.

C1. Quantification of nanoparticle mass taken up by macrophages

Experimental Outline

We reasoned that since MSNs take up various compounds depending on the physical and chemical properties of the compounds, each MSN, given a defined MSN preparation and a cargo compound, must take up comparable amounts of the cargo compound. Therefore, if MSNs are loaded with a dye, increasing number of MSNs should lead to a linear increase in the fluorescence intensity of the dye that is loaded on the MSNs. Next, we tested this hypothesis with following experiment: We prepared various amounts of Dil-filled MSNs (1000, 500, 250, 125, 62.5, 31.25, 15.62, 7.81 µg/ml) in 100µl PBS (using a 96 Well Black/Clear Bottom Plate, TC Surface, 165305, Thermo Scientific) and measured the corresponding fluorescence intensities (Ex/Em: 530/580 nm) (BioTek Cytation 7 microplate reader, Agilent). As shown in Figure S12, we found a linear relationship (R^2 =1, coefficient of determination) between the concentration of Dil-filled MSNs and fluorescence intensity upon linear regression fit. We used this strategy to quantify the amount of Dil-filled MSNs loaded on macrophages. 50µg Dil-filled MSNs were loaded on $5x10^5$ BMDMs. Of these, $2.5x10^5$ cells in 100µl PBS were used for fluorescence intensity measurement. These intensities were then converted to amount of Dil-filled MSNs using the linear regression.

Using the equation of the linear regression, we found that $2.5x10^5$ cells were loaded with Dil-filled MSNs corresponding to an amount of 15.275 µg. Therefore, 30.55 µg MSNs were taken up by $5x10^5$ BMDMs, which corresponds to an uptake efficiency of 61%. From this, considering a distribution of MSN cellular uptake, we could calculate the average amount of MSNs taken up by each macrophage to be 61pg.

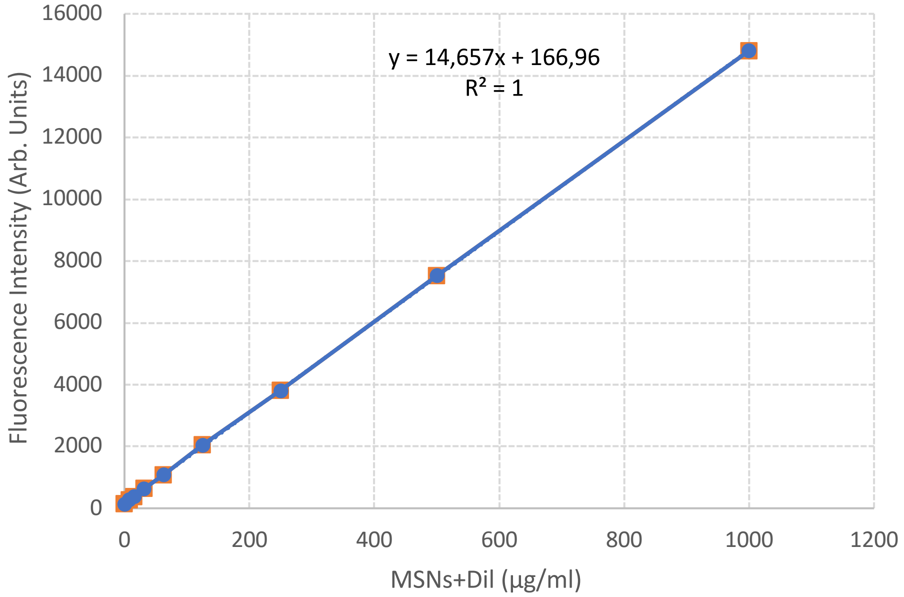

Figure S12: Linear regression fit between concentration of Dil-filled MSNs and corresponding fluorescence intensity. The equation represents the linear regression. The coefficient of determination (R^2) is found to be 1 indicating a perfect linear relationship between the two variables.

C2. qRT-PCR analysis of M1/M2 markers upon loading with ZrO-CLP-NPs

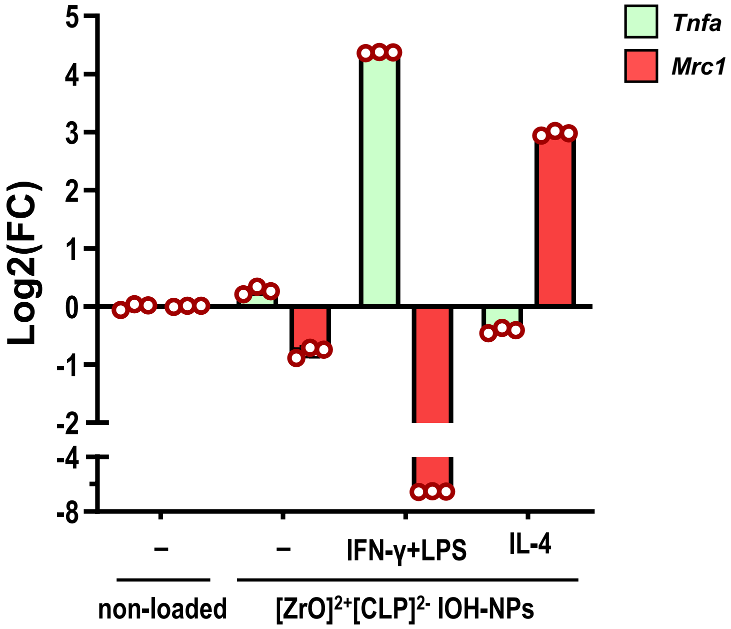

Figure S13: BMDMs were loaded with ZrO-CLP-NPs and subsequently cultivated for 24 hours either in standard culture conditions or in the presence of IFN-γ (50 ng/ml) and LPS (100 ng/ml) or in the presence of IL-4 (20 ng/ml). Non-loaded BMDMs were incubated for 24 hours with control medium. mRNA expression of M1 (*Tnfa*) and M2 (*Mrc1*) markers was determined by RT-qPCR and normalized to beta-actin expression. Results are expressed as means S.D.

C3. Quantification of type I/III IFN production by loaded macrophages

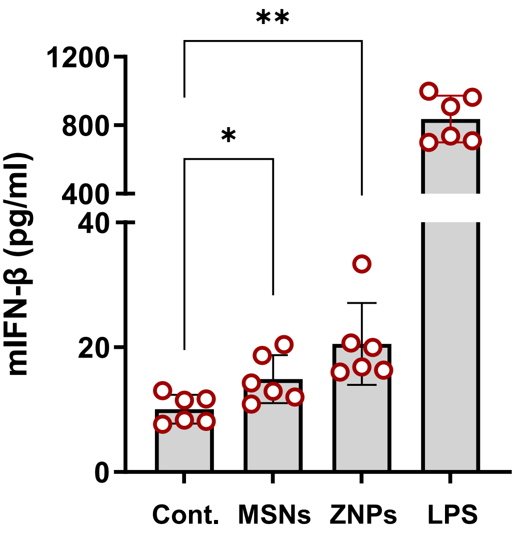

Figure S14: J774a.1 cells were loaded with MSNs or ZrO-CLP-NPs (ZNPs) and subsequently cultivated for 24 hours in standard culture conditions. Non-loaded cells or cells stimulated with LPS (10 ng/ml) act as controls. Supernatant was collected, centrifuged and dilutions were added to NIH3T3 Mx2-Luc reporter cells for 24h. Cells were lysed in reporter lysis buffer (RLB, E4030 Promega) and luciferase assay was performed using Berthold Technologies Lumat LB 9507 luminometer. Total protein was quantified using Peqlab Spectrophotometer ND-1000 Nanodrop. RLU values were normalized using protein measurements to generate RLU/mg Protein value. Final mIFN-beta (pg/ml) values were calculated from a murine IFN-beta standard curve and linear regression analysis (6.25, 12.5, 25, 50, 100, 200 pg/ml IFN-beta; Sino Biological, #50708). Values represent means ± S.D. (n=6). * = $P<0.05$, ** = $P<0.01$, unpaired t-test.

NIH3T3 Mx2-Luc cells were derived from NIH3T3 cells by transfection of a bacterial artificial chromosome (BAC RP24-71I6) encoding luciferase under the control of the murine interferon-stimulated Mx2 promoter [S4]. This system is capable of detecting murine type I IFN activity with a sensitivity of 5pg/ml. NIH3T3 Mx2-Luc cells were cultured in DMEM supplemented with 10% FBS, 1 mM sodium pyruvate solution (Sigma-Aldrich), 10 mM HEPES, 1× MEM, 0.05 mM beta-mercaptoethanol, 60 µg/mL penicillin, and 100 µg/mL streptomycin.

C4. Quantification of 4-OHT based on Cre-luc reporter cells

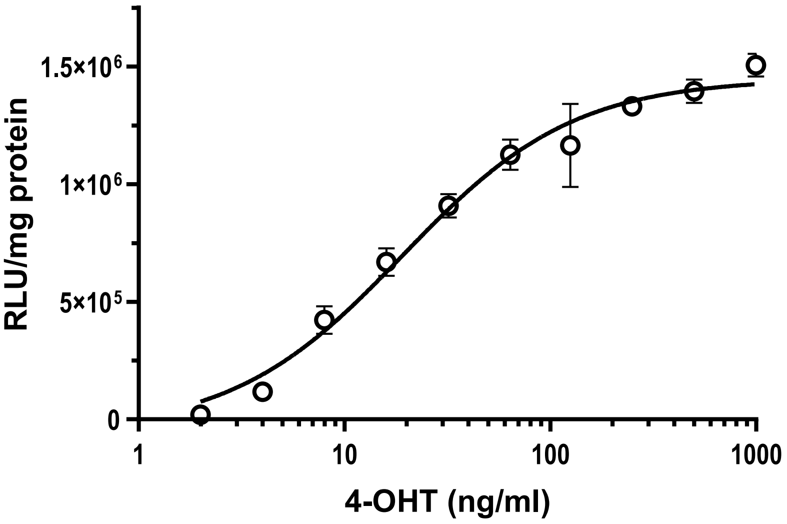

Figure S15: 4-OHT response curve on Cre-luc reporter cells. $3x10^4$ cells were seeded in 48 well plate and cultured overnight. 4-OHT was diluted in standard culture medium to indicated concentrations (2ng/ml to 1000ng/ml) and reporter cells were stimulated for 24h before harvesting for luciferase assay.

D. References

[S1] (a) M. Ischyropoulou, K. Sabljo, L. Schneider, C. M. Niemeyer, J. Napp, C. Feldmann, F. Alves, High-Load Gemcitabine Inorganic-Organic Hybrid Nanoparticles as Image-Guided Tumor-Selective Drug-Delivery System To Treat Pancreatic Cancer. *Adv. Mater.* **2023**, *35*, 2305151(1-15).
(b) B. L. Neumeier, M. Khorenko, F. Alves, O. Goldmann, J. Napp, U. Schepers, H. M. Reichardt, C. Feldmann, Fluorescent Inorganic-Organic Hybrid Nanoparticles. *ChemNanoMat* **2019**, *5*, 24-45.
(c) J. G. Heck, J. Napp, S. Simonato, J. Möllmer, M. Lange, H. R. Reichardt, R. Staudt, F. Alves, C. Feldmann, Multifunctional Phosphate-based Inorganic-Organic Hybrid Nanoparticles. *J. Am. Chem. Soc.* **2015**, *137*, 7329-7336

[S2] J. G. Heck, K. Rox, H. Lünsdorf, T. Lückerath, N. Klaassen, E. Medina, O. Goldmann, C. Feldmann, Zirconyl Clindamycin Phosphate Antibiotic Nanocarriers for Targeting Intracellular Persisting Staphylococcus aureus. *ACS Omega* **2018**, *3*, 8589-8594.

[S3] Nandiyanto, A.B.D., Kim, S.-G., Iskandar, F., and Okuyama, K. (2009). Synthesis of spherical mesoporous silica nanoparticles with nanometer-size controllable pores and outer diameters. Microporous and Mesoporous Materials, 120 (3), 447-453. doi.org/10.1016/j.micromeso.2008.12.019.

[S4] Pulverer JE, Rand U, Lienenklaus S, Kugel D, Zietara N, Kochs G, et al. Temporal and spatial resolution of type I and III interferon responses in vivo. Journal of virology. 2010;84(17):8626-38.
